# Potent and broadly neutralizing antibodies against sarbecoviruses induced by sequential COVID-19 vaccination

**DOI:** 10.1038/s41421-024-00648-1

**Published:** 2024-02-06

**Authors:** Xiaoyu Zhao, Tianyi Qiu, Xiner Huang, Qiyu Mao, Yajie Wang, Rui Qiao, Jiayan Li, Tiantian Mao, Yuan Wang, Yewei Cun, Caicui Wang, Cuiting Luo, Chaemin Yoon, Xun Wang, Chen Li, Yuchen Cui, Chaoyue Zhao, Minghui Li, Yanjia Chen, Guonan Cai, Wenye Geng, Zixin Hu, Jinglei Cao, Wenhong Zhang, Zhiwei Cao, Hin Chu, Lei Sun, Pengfei Wang

**Affiliations:** 1 Shanghai Pudong Hospital, Fudan University Pudong Medical Center, State Key Laboratory of Genetic Engineering, MOE Engineering Research Center of Gene Technology, School of Life Sciences, Shanghai Institute of Infectious Disease and Biosecurity, Institutes of Biomedical Sciences, Shanghai Sci-Tech Inno Center for Infection & Immunity, Fudan University, Shanghai, China; 2 Institute of Clinical Science, ZhongShan Hospital, Shanghai Institute of Infectious Disease and Biosecurity, Fudan University, Shanghai, China; 3 Department of Microbiology, School of Clinical Medicine, Li Ka Shing Faculty of Medicine, The University of Hong Kong, Pokfulam, Hong Kong China; 4 Shanghai Fifth People’s Hospital, Fudan University, Shanghai, China; 5 School of Life Sciences, Fudan University, Shanghai, China; 6 School of Life Sciences and Technology, Tongji University, Shanghai, China; 7 Fudan Zhangjiang Institute, Shanghai Medical College of Fudan University, Fudan University, Shanghai, China; 8 Artificial Intelligence Innovation and Incubation Institute, Fudan University, Shanghai, China; 9 Department of Infectious Diseases, Shanghai Key Laboratory of Infectious Diseases and Biosafety Emergency Response, National Medical Center for Infectious Diseases, Huashan Hospital, Fudan University, Shanghai, China

**Keywords:** Mechanisms of disease, Autoimmunity

## Abstract

The current SARS-CoV-2 variants strikingly evade all authorized monoclonal antibodies and threaten the efficacy of serum-neutralizing activity elicited by vaccination or prior infection, urging the need to develop antivirals against SARS-CoV-2 and related sarbecoviruses. Here, we identified both potent and broadly neutralizing antibodies from a five-dose vaccinated donor who exhibited cross-reactive serum-neutralizing activity against diverse coronaviruses. Through single B-cell sorting and sequencing followed by a tailor-made computational pipeline, we successfully selected 86 antibodies with potential cross-neutralizing ability from 684 antibody sequences. Among them, PW5-570 potently neutralized all SARS-CoV-2 variants that arose prior to Omicron BA.5, and the other three could broadly neutralize all current SARS-CoV-2 variants of concern, SARS-CoV and their related sarbecoviruses (Pangolin-GD, RaTG13, WIV-1, and SHC014). Cryo-EM analysis demonstrates that these antibodies have diverse neutralization mechanisms, such as disassembling spike trimers, or binding to RBM or SD1 to affect ACE2 binding. In addition, prophylactic administration of these antibodies significantly protects nasal turbinate and lung infections against BA.1, XBB.1, and SARS-CoV viral challenge in golden Syrian hamsters, respectively. Importantly, post-exposure treatment with PW5-5 and PW5-535 also markedly protects against XBB.1 challenge in these models. This study reveals the potential utility of computational process to assist screening cross-reactive antibodies, as well as the potency of vaccine-induced broadly neutralizing antibodies against current SARS-CoV-2 variants and related sarbecoviruses, offering promising avenues for the development of broad therapeutic antibody drugs.

## Introduction

As of Dec 31, 2023, severe acute respiratory syndrome coronavirus 2 (SARS-CoV-2), the causative agent of coronavirus disease 2019 (COVID-19), has caused over 773 million confirmed cases, along with more than 6.9 million deaths worldwide (https://covid19.who.int). During the course of COVID-19 pandemic, SARS-CoV-2 continuously evolves with changes in the genome caused by genetic mutations or viral recombination, resulting in variants that are different from the original SARS-CoV-2 virus^1–3^. The World Health Organization has now designated five SARS-CoV-2 variants of concern (VOCs), including Alpha (B.1.1.7), Beta (B.1.351), Gamma (P.1), Delta (B.1.617.2), and Omicron (B.1.1.529 or BA.1)^4^. In particular, the Omicron VOC contains an alarming number of over 30 mutations in its spike (S) protein and has spread rapidly worldwide^5^. This situation continues to evolve with increased global spreading of Omicron subvariants, including BA.2, BA.2.12.1, BA.2.75, BA.2.75.2, BA.4, BA.4.6, BA.5, BF.7, BN.1, BQ.1, BQ.1.1, CH.1.1, and the most recent XBB sublinages^6–9^.

Although COVID-19 vaccines and therapeutic antibodies have been developed and deployed at an unprecedented speed, the continued evolution of SARS-CoV-2 variants has raised concerns about the effectiveness of monoclonal antibody (mAb) therapies and the potential evasion from vaccine-induced immunity^10–12^. Recent studies have revealed that the efficacy of mRNA monovalent vaccine after two or three doses against severe infection or death is relatively high, while the induction of cross-neutralizing antibodies against recent Omicron variants infection was less than 50% during the BA.4/5 waves, and even a fourth dose only caused a minimal transient increase in Omicron-neutralizing antibodies^10,13,14^. In addition, BQ and XBB subvariants have been reported to have an immune evasion capacity higher than BA.5 in vaccinated patients^15–17^. We and other groups have also shown that two doses of an inactivated whole-virion vaccine showed weak to no neutralization activity, although homologous or heterologous boosters improved neutralization titers against Omicron subvariants^4,18,19^. Moreover, several approved and clinical-stage mAbs suffer from a loss of efficacy against Omicron subvariants^20–22^. In particular, the Omicron subvariants BQ.1.1 and XBB.1.5 have resulted in marked or complete resistance to neutralization of almost all authorized antibodies^9^. Considering that the authorized or approved mAbs for clinical immunotherapy have shown greatly reduced activities, it is necessary to identify broadly neutralizing antibodies that fully cover the various SARS-CoV-2 variants.

Apart from SARS-CoV-2, SARS-CoV that use human angiotensin-converting enzyme 2 (ACE2) as receptor, and Middle East respiratory syndrome coronavirus (MERS-CoV) belong to beta-coronaviruses using receptors other than ACE2, have previously posed a great threat to human health^23,24^. In addition, the growing threat of continued zoonotic spillovers reveals the importance of the development of interventions that could broadly combat zoonotic coronaviruses with pandemic potential^25,26^. For instance, a previous study reported that a SARS-CoV-2-related pangolin coronavirus exhibits similar infection characteristics to SARS-CoV-2 and can direct contact transmissibility in hamsters^27^. More recently, another pangolin-origin SARS-CoV-2-related coronavirus also showed similar infectivity to SARS-CoV-2 in both human cells and organoids, highlighting the potential risk of spillover from pangolins and follow-up circulation in human populations^28^. Therefore, finding potent and broadly neutralizing mAbs that can target not only the circulating SARS-CoV-2 variants but also related sarbecoviruses is of utmost importance.

In this study, we reported the isolation of potent and broadly neutralizing mAbs from a vaccinated donor with a special five-dose COVID-19 vaccination schedule. One mAb, named PW5-570, displayed the most potent neutralizing activity against all SARS-CoV-2 strains prior to BA.5 variant, and another three mAbs, named PW5-4, PW5-5, and PW5-535 were found to broadly neutralize all sarbecoviruses tested, including all the current SARS-CoV-2 variants, SARS-CoV, Pangolin-GD, RaTG13, WIV-1, and SHC014. Structural analysis showed that these antibodies had a variety of binding modes, with PW5-5 and PW5-535 binding to different conserved epitopes hidden in receptor-binding domain (RBD), while the epitope of PW5-570 overlapped with receptor-binding motif (RBM) to affect binding to ACE2. Moreover, prophylactic administration of PW5-570 significantly protected nasal turbinate and lung infections against Omicron BA.1 challenge in golden Syrian hamsters, displaying promising biomedical interventions against pandemic SARS-CoV-2 VOCs. More importantly, PW5-5 and PW5-535 exhibited in vivo efficacy against both SARS-CoV-2 XBB.1 and SARS-CoV, suggesting their use as pan-sarbecovirus therapeutic antivirals. Taken together, from a single sequential vaccinated donor, we not only found an ultra-potent neutralizing mAb against SARS-CoV-2 VOCs, but also identified three broadly neutralizing mAbs with pan-sarbecovirus potential. This study reveals the potency of vaccine-induced broadly neutralizing mAbs against current VOCs and other related sarbecoviruses, and affords the potential for broad therapeutic mAb drugs.

## Results

### Identification of a sequential vaccinated donor with cross-reactive serum-neutralizing activity

**Fig. 1:**
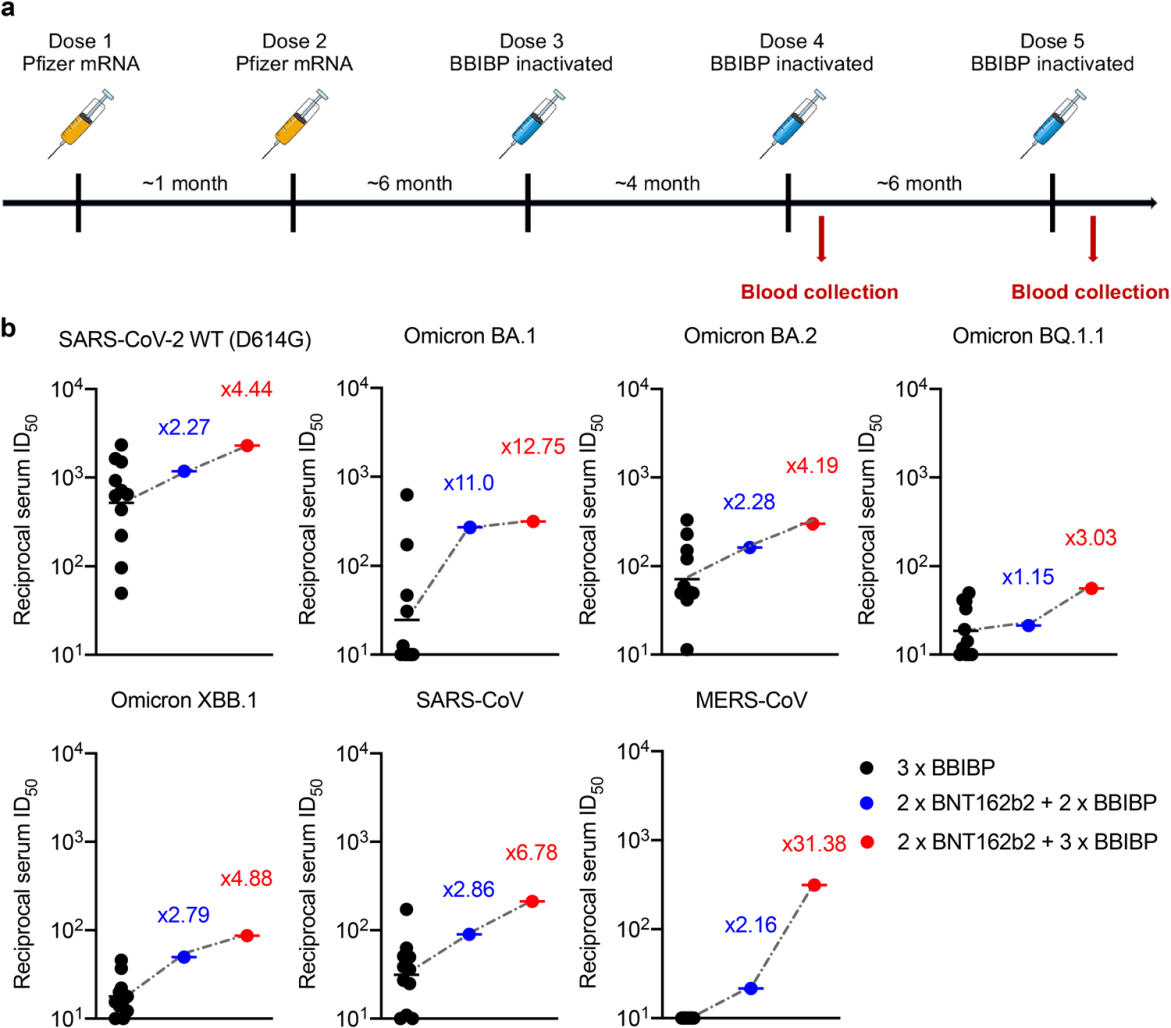
Cross-reactive immune responses elicited by sequential vaccination. **a** Vaccination schedule overview. A healthy volunteer received a total of five doses of vaccine, starting with a two-dose mRNA vaccine (BNT162b2) followed by a three-dose inactivated whole-virion vaccine (BBIBP-CorV). Blood samples were collected after the 4th and 5th vaccination dose as illustrated in the diagram. **b** Neutralizing antibody titers of the sequential vaccination doner (4-dose: blue and 5-dose: red) compared to the 3-dose BBIBP vaccinees (black, *n* = 11). Neutralizing antibody titers represent serum dilution required to achieve 50% virus neutralization (ID_50_). The numbers indicate the fold of enhancement of ID_50_ values relative to mean titer measured among 3-dose BBIBP vaccinees.

To isolate potent broadly neutralizing mAbs against currently circulating SARS-CoV-2 variants and other human coronaviruses, we screened out one healthy volunteer with no history of exposure to SARS-CoV-2, who had received a total of five COVID-19 vaccination doses, starting with two doses of mRNA vaccine (BNT162b2) followed by three doses of inactivated whole-virion vaccine (BBIBP-CorV), as depicted in Fig. 1a. We collected serum samples from this donor after the fourth and fifth vaccination, and tested their neutralizing antibody titer (ID_50_) against a panel of pseudotyped human coronaviruses, including SARS-CoV-2 wild-type (WT, D614G), BA.1, BA.2, BQ.1.1, and XBB.1 variants, as well as SARS-CoV and MERS-CoV. For comparison, neutralization ID_50_ values were also measured for 11 local vaccinees, whose blood samples were collected at 1 month after the third BBIBP-CorV-vaccination (Fig. 1b; Supplementary Fig. S1). Although the Omicron subvariants showed substantial neutralization evasion from all the serum samples, similar to what we reported previously^17^, serum from the sequential vaccination donor after the fourth and fifth dose had consistently higher neutralization titers than the three-dose inactivated vaccination group. Particularly, the serum after the fifth vaccination dose reached 3–13-fold higher neutralization titers than the mean of the three-dose inactivated vaccination group against SARS-CoV-2 and its variants. In addition, the serum after the fifth dose also showed a sevenfold higher SARS-CoV neutralization titer than the mean of the three-dose inactivated vaccination group. More importantly, the four- and five-dose sequential COVID-19 vaccination even elicited cross-reactive neutralizing antibodies against MERS-CoV, but not the three-dose homologous inactivated vaccination, which induced undetectable neutralization activity against MERS-CoV (ID_50_ < 1:10) (Fig. 1b). These results indicated that sequential immunization of heterologous COVID-19 vaccinations could elicit cross-reactive broadly neutralizing antibodies against SARS-CoV-2, its variants, SARS-CoV, and even MERS-CoV. We, therefore, chose this special vaccinee for subsequent search of broadly neutralizing mAbs.

### Identification of broadly neutralizing antibodies against severe human coronaviruses

**Fig. 2:**
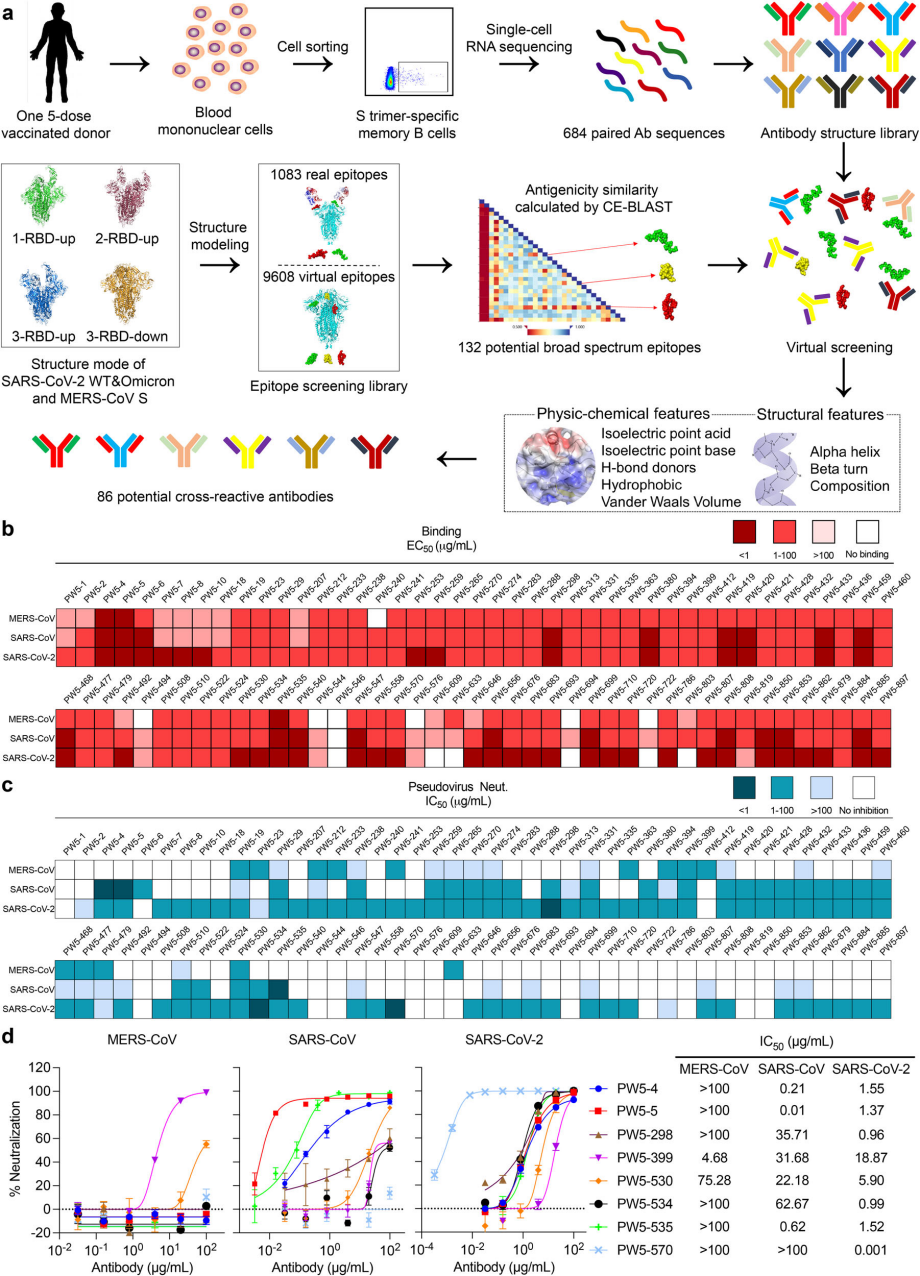
Identification of broadly neutralizing antibodies against severe human coronaviruses. **a** Schematic representation of the isolation process for potential broadly neutralizing antibodies using a combined method of single B-cell sorting and virtual screening. **b** Heatmap showing the binding EC_50_ values of the selected mAbs with S trimers of MERS-CoV, SARS-CoV, and SARS-CoV-2, respectively. **c** Heatmap showing the neutralization IC_50_ values of each mAb with indicated pseudotyped viruses. **d** Neutralization curves (left) and IC_50_ values (right) of indicated mAb with MERS-CoV, SARS-CoV, and SARS-CoV-2 pseudotyped viruses, respectively. The data are representative of one of at least three independent experiments and are presented as the mean ± SEM.

Peripheral blood mononuclear cell (PBMC) samples were collected from this selected donor 2 weeks following the fifth immunization dose after informed consent. We then sorted for MERS-CoV or SARS-CoV-2 Omicron S trimer-specific memory B cells from the blood (Supplementary Fig. S2), followed by single-cell RNA sequencing (scRNA-seq) to determine the paired heavy and light chain sequences of each mAb. Subsequently, an in silico screening was performed to identify potential cross-reactive mAbs for both MERS-CoV and SARS-CoV-2. Initially, the structure modeling of S protein from three coronaviruses (MERS-CoV, SARS-CoV-2 WT, and SARS-CoV-2 Omicron), each including four structure formats (1-RBD-up, 2-RBD-up, 3-RBD-up and 3-RBD-down) (Supplementary Fig. S3), and the 684 antibodies were constructed. Then, we screened 1083 real epitopes and 9608 virtual epitopes of the coronavirus S proteins through the conformational epitope-basic local alignment search tool (CE-BLAST)^29^, which identified 132 potential cross-reactive epitopes. Finally, through the patch model of SEPPA-mAb^30^, we ranked the binding score between each cross-reactive epitope and the complementarity-determining region (CDR) of the antibodies through structure complementarity calculation. Eventually, 86 top-ranking mAbs for at least one cross-reactive epitope were isolated for further analysis (Fig. 2a).

To determine whether there were differences between the selected 86 mAbs and the total 684 mAbs, we characterized their heavy and light chain V gene usage, CDR3 length, and the number of nucleotide mutations. The results showed that apart from the same V gene usage, IGHV3-11, IGHV3-21, IGKV2D-28, IGKV 2-30, IGLV3-1, IGLV2-11, and IGLV3-25 were enriched in the selected 86 mAbs compared with the 684 mAbs (Supplementary Fig. S4a, b). For the CDR3 length, no significant difference was observed between the 86 mAbs and the 684 mAbs, and the peak of the heavy chain and light chain is about 16 aa and 11 aa, respectively (Supplementary Fig. S4c, d). Notably, the number of nucleotide mutations in the 86 mAbs is significantly higher than that in the 684 mAbs, indicating that the selected 86 mAbs have a more violent mutation than the rest of the antibodies in the 684 mAbs (Supplementary Fig. S4e, f).

To determine the cross-binding abilities of the 86 mAbs, we assayed the half-maximal binding concentration (EC_50_) values of these mAbs to the S trimer of MERS-CoV, SARS-CoV and SARS-CoV-2, respectively. We found that most of the mAbs were highly cross-reactive to the S trimers of SARS-CoV and SARS-CoV-2 but not to that of MERS-CoV, whereas PW5-4, PW5-5 and PW5-535 displayed high binding ability to all of them (Fig. 2b). We further assayed the cross-activities of those mAbs against MERS-CoV, SARS-CoV and SARS-CoV-2 by vesicular stomatitis virus (VSV)-based pseudotyped virus neutralization. Consistent with the binding profiles for these mAbs, most of the mAbs showed cross-neutralizing activity to SARS-CoV and SARS-CoV-2 but less to MERS-CoV (Fig. 2c). Among them, PW5-4, PW5-5, PW5-298, PW5-534, and PW5-535 showed good neutralizing activity against both SARS-CoV and SARS-CoV-2, while PW5-399 and PW5-530 displayed better broadly neutralizing potential, covering all three severe human coronaviruses including MERS-CoV. Another mAb PW5-570 only showed neutralizing activity against SARS-CoV-2, but its activity was ultra-potent with a half-maximal inhibitory concentration (IC_50_) of 1 ng/mL. The pseudovirus neutralization profiles for these selected 8 mAbs were shown in Fig. 2d. These results demonstrated that ultra-potent SARS-CoV-2 neutralizing antibodies, as well as broadly neutralizing antibodies against other human coronaviruses, could be elicited by sequential SARS-CoV-2 vaccination.

### Characterizing the potency and breadth of neutralization conferred by selected antibodies

**Fig. 3:**
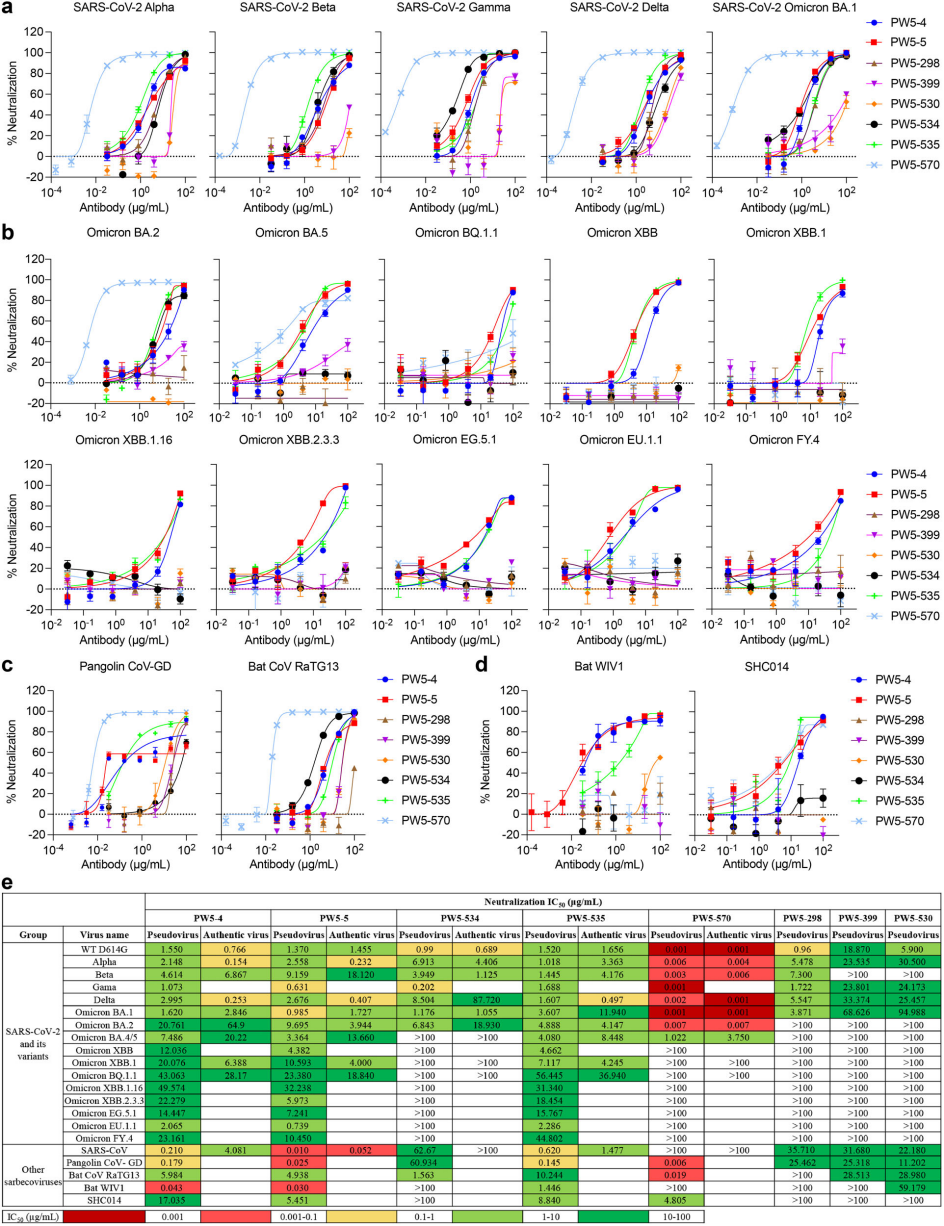
Neutralization of pseudotyped and authentic SARS-CoV-2 variants, SARS-CoV and other sarbecoviruses. **a** Neutralization of SARS-CoV-2 variants of concern (Alpha, Beta, Gamma, Delta, and Omicron BA.1) by indicated mAb. **b** Neutralization of major SARS-CoV-2 Omicron subvariants, including BA.2, BA.5, BQ.1.1, and XBB, as well as its latest subvariants, such as XBB.1, XBB.1.16, XBB.2.3.3, EG.5.1, EU.1.1, and FY.4 by indicated mAb. **c** Neutralization of SARS-CoV-2-related sarbecoviruses (Pangolin CoV-GD and Bat CoV RaTG13) by indicated mAb. **d** Neutralization of SARS-CoV-related sarbecoviruses (Bat WIV-1 and SHC014) by indicated mAb. **e** IC_50_ neutralization values of each mAb against SARS-CoV-2 and its variants, and other related sarbecoviruses pseudoviruses as well as the indicated live viruses. The data are representative of one of at least three independent experiments and are presented as the mean ± SEM.

To understand the breadth of these 8 newly cloned mAbs, we first performed neutralization assays using pseudotyped SARS-CoV-2 VOC viruses, including Alpha, Beta, Gamma, Delta, and Omicron (BA.1) (Fig. 3a, e). All mAbs, except PW5-399 and PW5-530, showed neutralization breadth against all SARS-CoV-2 VOCs. PW5-570 was the most potent with extremely low IC_50_ values, followed by a group composed of PW5-4, PW5-5, PW5-298, PW5-534, and PW5-535. We next evaluated each mAb for their neutralization against major SARS-CoV-2 Omicron subvariants, including BA.2, BA.5, BQ.1.1, and XBB, as well as its latest subvariants, such as XBB.1, XBB.1.16, XBB.2.3.3, EG.5.1, EU.1.1, and FY.4 (Fig. 3b, e). Only PW5-4, PW5-5, and PW5-535 neutralized all Omicron subvariants tested, whereas other mAbs showed reduced or abolished neutralizing activity against certain viruses in the panel. In addition, we evaluated each mAb for neutralization against four SARS-CoV- or SARS-CoV-2-related sarbecoviruses that are capable of using human ACE2 as a receptor. We found that all mAbs, except PW5-298, neutralized the two SARS-CoV-2-related sarbecoviruses (Pangolin-GD and RaTG13), and PW5-570 displayed the most potent neutralizing activity (Fig. 3c, e). In contrast, for SARS-CoV-related sarbecoviruses (WIV-1 and SHC014), only PW5-4, PW5-5 and PW5-535 retained neutralizing activity, but not the other mAbs (Fig. 3d, e).

We next performed authentic virus neutralization assays in Vero-E6 cells for five selected mAbs (PW5-4, PW5-5, PW5-534, PW5-535, and PW5-570) with better neutralization activity (Fig. 3e). Similar to the pseudovirus results, we consistently found that PW5-4, PW5-5 and PW5-535 showed breadth by neutralizing all tested SARS-CoV and SARS-CoV-2 variants. Meanwhile, PW5-570 was the most potent mAb for all SARS-CoV-2 strains prior to the Omicron BA.5 variant. Besides, the neutralizing titers against the pseudoviruses correlated quite well with the titers obtained against the live virus (Supplementary Fig. S5). Taken together, these results demonstrated that PW5-570 potently neutralized all SARS-CoV-2 strains prior to the Omicron BA.5 variant, but with no cross-neutralizing activity against SARS-CoV. While PW5-4, PW5-5 and PW5-535 neutralized all the tested SARS-CoV-2 variants as well as SARS-CoV and other related sarbecoviruses, PW5-5 exhibited better potency than the other two.

To further characterize these selected antibodies and investigate the potential for antibody combination, we measured the binding affinity and examined the competition between the mAbs and other four well-defined mAbs (CB6, LY-CoV555, S309, and CR3022) using the enzyme-linked immunosorbent assay (ELISA)^31–34^. We found that all five selected antibodies had a high affinity for the RBD but not the N-terminal domain (NTD) of the S protein (Supplementary Fig. S6a). Besides, the competition profile indicated both overlapping and distinct epitopes recognized by these antibodies (Supplementary Fig. S6b). PW5-4 and PW5-5 with overlapping epitopes, as well as the PW5-535, competed with the CR3022 (Class IV) for RBD binding. This analysis additionally revealed that through steric hindrance or binding-induced conformational change in RBD, PW5-570 is at least partially competitive with both CB6 (Class I) and LY-CoV555 (Class II), while PW5-534 is competitive with both LY-CoV555 (Class II) and S309 (Class III). We next performed a competition assay on S binding between the antibodies and ACE2 to investigate the neutralizing mechanism of these antibodies. As shown in Supplementary Fig. S6c, d, the binding of ACE2 to SARS-CoV-2 S trimer was suppressed when PW5-534, PW5-535, and PW5-570 were mixed with SARS-CoV-2 S trimer, indicating a competition between these antibodies and ACE2. Thus, these results suggested that sequential vaccination could induce a broad spectrum of antibodies with distinct epitopes similar to those following natural infection.

### Cryo-EM structure of PW5-570 complexed with Omicron BA.1 S trimer

**Fig. 4:**
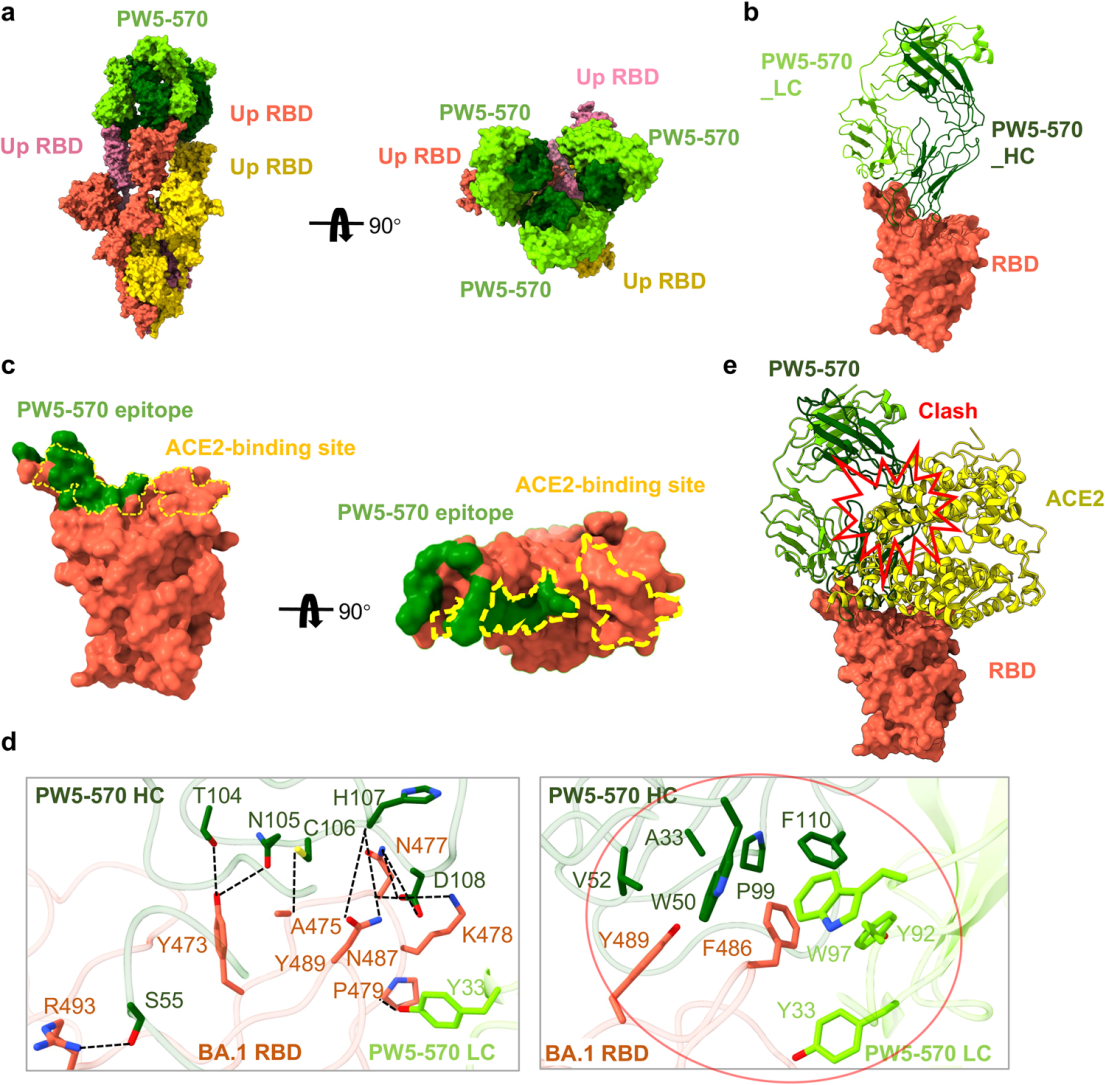
Cryo-EM structure of PW5-570 IgG with Omicron BA.1 S trimer. **a** Cryo-EM structures of the BA.1 S trimer in complex with the antibody PW5-570 IgG. **b** Structure of BA.1-RBD–PW5-570. The RBD is displayed in tomato surface mode. The heavy chain and light chain of PW5-570 are shown as ribbons colored in dark green and light green, respectively. **c** Close-up view of the PW5-570 epitope on the RBD. ACE2-binding epitopes are represented by yellow dashed lines. **d** Detailed interactions between the PW5-570 and BA.1 S RBD. Hydrogen bonds are represented by dashed lines. Hydrophobic interactions are marked with red circles. **e** PW5-570 clash with the ACE2. ACE2 is marked yellow.

To understand the mechanism of SARS-CoV-2 Omicron variants potently neutralized by PW5-570, we determined the structure of Omicron BA.1 S trimer in complex with PW5-570 immunoglobulin G (IgG) using cryo-electron microscopy (cryo-EM) (Fig. 4a). The purified BA.1 S trimer was mixed with PW5-570 at a 1:1.2 molar ratio, incubated at 4 °C for 1 h, and further purified by gel filtration. The peak fraction of the gel filtration was used for cryo-EM data collection (Supplementary Fig. S7a). Unsymmetrical trimer dimers cross-linked by crystallizable fragment (Fc) regions were observed in 2D classification (Supplementary Fig. S15). S trimers with 3 RBDs up, each binding to one PW5-570 fragment of antigen binding (Fab), were solved at 2.78 Å resolution. The Fc region is invisible in the final structure because of its flexibility. The S RBD with PW5-570 Fab region was locally refined to 2.93 Å.

Structure analysis shows that the epitope of PW5-570 mostly overlaps with the RBM (Fig. 4b, c). The binding of PW5-570 to RBD covers a surface area of 793 Å^2^ and involves a total of 12 RBD residues (Fig. 4d). S55 of CDRH2 and T104, N105, C106, H107, D108 of CDRH3 contacts R493, Y473, A475, N487, N477, and K478 through 9 pairs of hydrogen bonds, and Y33 of CDRL1 forms a hydrogen bond with P479. In addition, A33 of CDRH1, W50, V52 of CDRH2, P99, F110 of CDRH3, Y33 of CDRL1, Y92, and W97 of CDRL3 form a hydrophobic interface with F486 and Y489. The binding epitopes of PW5-570 to RBD include the key residues Y473, A475, F486, and N487, required for ACE2 recognition and binding. Superposing the structures of the RBD/PW5-570 and RBD/ACE2 complexes showed extensive clashes between the antibody and the ACE2 receptor (Fig. 4e), suggesting that binding of PW5-570 to RBD produces steric hindrance and prevents S from binding to the receptor ACE2. This agrees with the RBD–ACE2 blocking capacity of PW5-570 (Supplementary Fig. S6c, d).

To further verify the key mutations conferring PW5-570 resistance, we constructed pseudoviruses with each of the single mutations alone or in combination based on Omicron BA.2 S. Consistent with cryo-EM structure of PW5-570, F486V is indeed the key mutation responsible for the loss in potency of PW5-570, while R493Q has the effect of mitigating its escape (Supplementary Fig. S8).

### Cryo-EM structure of PW5-5 complexed with Omicron XBB and SARS-CoV S trimer

To understand the broad neutralization mechanism of PW5-5, we determined the cryo-EM structure of PW5-5 complexed with the stabilized prefusion ectodomain of Omicron XBB S (6 P) and SARS-CoV S (2 P), respectively. The purified XBB S trimer was mixed with PW5-5 IgG at a 1:1.2 molar ratio, incubated at 4 °C for 1 h, and further purified by gel filtration. The peak fraction of the gel filtration was used for negative-staining EM imaging, showing that PW5-5 binding disassembled XBB S trimer (Supplementary Fig. S9). Therefore, we shortened the incubation time to 15 min and frozen the sample directly without further purification. The same strategy was used for SARS-CoV S–PW5-5.

**Fig. 5:**
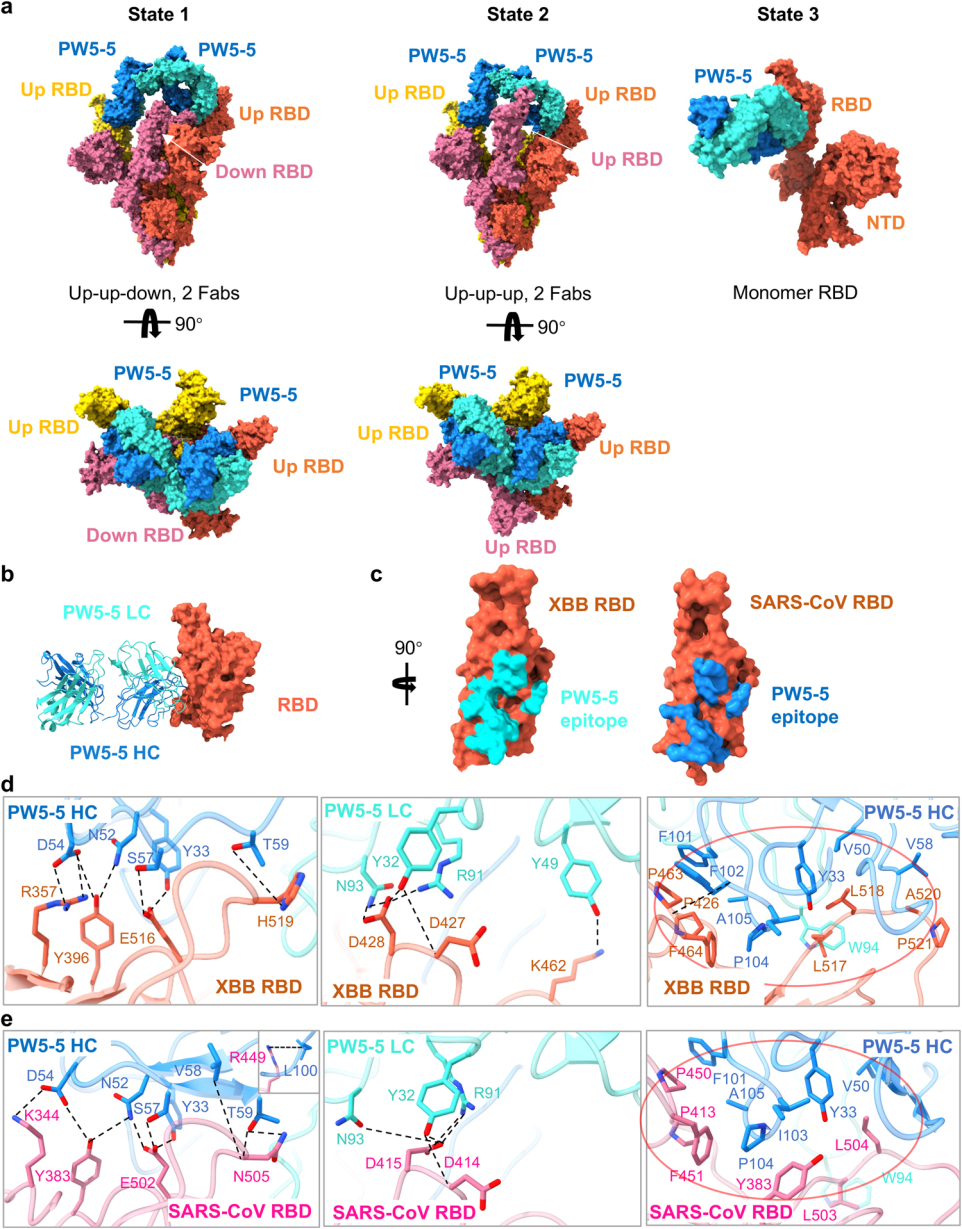
Cryo-EM structure of PW5-5 IgG with Omicron XBB and SARS-CoV S trimer. **a** Cryo-EM structures of the XBB S in complex with the antibody PW5-5 IgG. **b** Structure of XBB RBD–PW5-5. The RBD is displayed in tomato. The heavy chain and light chain of PW5-5 are shown as ribbons colored in dark blue and light blue, respectively. **c** Close-up view of the PW5-535 epitope on XBB and SARS-CoV RBD. **d** Detailed interactions between the PW5-5 and XBB S RBD. **e** Detailed interactions between the PW5-5 and SARS-CoV S RBD. Hydrogen bonds are represented by dashed lines. Hydrophobic interactions are marked with red circles.

Cryo-EM characterization revealed three conformational states of XBB S–PW5-5 complex: the XBB S trimer with two up-RBDs binding two PW5-5 Fabs (state 1), the XBB S trimer with three up-RBDs binding two PW5-5 Fabs (state 2), and the XBB S monomer with one PW5-5 Fab (state 3), with resolution ranging from 3.1 Å to 3.7 Å (Fig. 5a; Supplementary Fig. S16). Thus, binding of IgG PW5-5 results in S trimer disassembly. The Fc region of PW5-5 is missing in the final structures because of its flexibility. The structure of monomer XBB S–PW5-5 was at sufficient resolution for model building. PW5-5 binds to a covert epitope inside the RBD, burying a 936 Å^2^ surface area (Fig. 5b, c). In total, 16 RBD residues are involved in the interaction. Among these, D54 of CDRH2 and R91 of CDRL3 form salt bridges with R357 and D428 of RBD, respectively. Y33 of CDRH1, N52, D54, S57, T59 of CDRH2, F102 of CDRH3, Y32 of CDRL1, Y49 of CDRL2 and N93 of CDRL3 form ten pairs of hydrogen bonds with E516, Y396, H519, P463, D427, D428 and K462 (Fig. 5d). In addition, Y33, V50, V58 of CDRH2, F101, F102, P104, A105 of CDRH3 and W94 of CDRL3 form a hydrophobic patch with F464, P426, P463, L517, L518, A520 and P521 of RBD. In SARS-CoV S–PW5-5 complex, two confirmations were observed: the monomer state and the 2 up-RBDs, 2 Fabs state (Supplementary Fig. S10a). The S RBD–PW5-5 Fab region was locally refined to 3.04 Å resolution (Supplementary Fig. S17). PW5-5 interacts with SARS-CoV RBD in a similar way, burying 978 Å^2^ surface area (Fig. 5e).

PW5-5 binding induces a large movement of the up-RBD: an outward rotation of 43.3° (up-RBD 1 in the 3-up state) and 55.9° (up-RBD 2 in the 3-up state) compared to the up-RBD in apo Omicron S (PDBID: 7WVN). This rotation is extraordinarily wider than previously reported antibodies FD01 (PDBID: 7WOQ) and bn03 (PDBID: 7WHK) with similar epitopes (Supplementary Fig. S10b)^35,36^. Such large deformation on S RBD may lead to instability. As no complex of SARS-CoV S trimer with 3 PW5-5 Fabs was observed, we propose that binding of the third Fab resulted in the disassembly of the S trimer. In summary, the binding of PW5-5 induces RBD into an extra-wide-open state, resulting in S trimer instability and disassembly.

### Cryo-EM structure of PW5-535 complexed with Omicron XBB and SARS-CoV S trimer

**Fig. 6:**
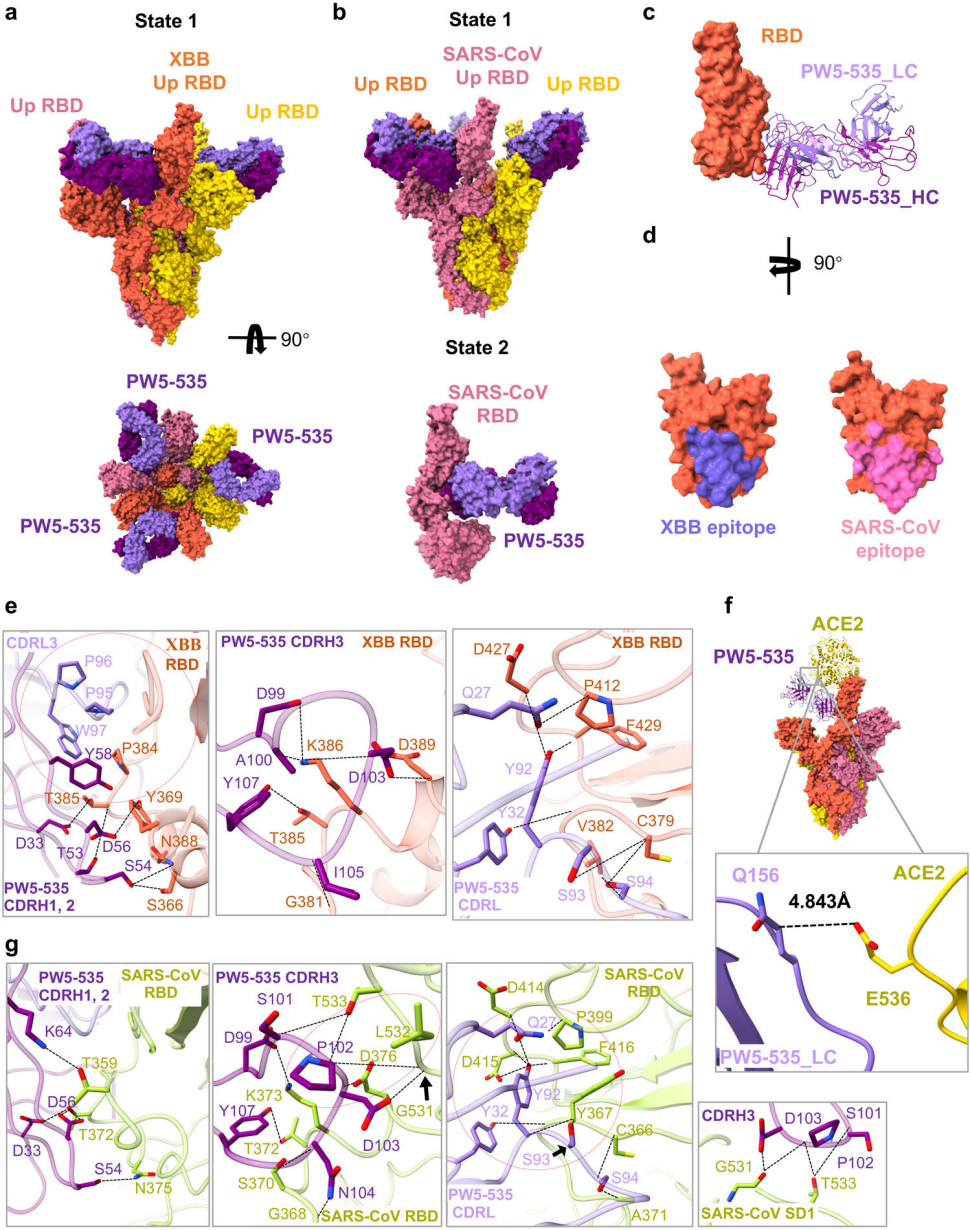
Cryo-EM structure of PW5-535 IgG with Omicron XBB and SARS-CoV S trimer. **a** Cryo-EM structure of the XBB S in complex with the antibody PW5-535 IgG. **b** Cryo-EM structures of the SARS-CoV S in complex with the antibody PW5-535 IgG. **c** Structure of XBB RBD–PW5-535. The RBD is displayed in tomato. The heavy chain and light chain of PW5-535 are shown as ribbons colored in dark purple and light purple, respectively. **d** Close-up view of the PW5-535 epitope on the XBB RBD and SARS-CoV RBD. **e** Detailed interactions between the PW5-535 and XBB S RBD. **f** PW5-535 causes steric hindrance with ACE2. **g** Detailed interactions between the PW5-535 and SARS-CoV S RBD and SD1. Hydrogen bonds are represented by dashed lines. Hydrophobic interactions are marked with red circles.

We also determined the structures of PW5-535 complexed with the stabilized prefusion ectodomain of Omicron XBB S (6 P) and SARS-CoV S (2 P). The same purification strategy as BA.1 S–PW5-570 was used for XBB S–PW5-535 and SARS-CoV S–PW5-535 (Supplementary Fig. S7b, c). Only one state of XBB S–PW5-535 particles was observed, which is 3 up-RBDs with 3 PW5-535 IgGs (Fig. 6a). The cryo-EM structures of 3 up-RBDs state were determined to 2.95 Å, with the RBD and PW5-535 region locally refined to 3.40 Å (Supplementary Fig. S18). As a contrast, two states of SARS-CoV S–PW5-535 were observed: 3 up-RBDs with 3 PW5-535 Fabs (3.05 Å), and RBD monomer with PW5-535 Fabs (3.54 Å) (Fig. 6b; Supplementary Fig. S19). Notably, the monomer state is only observed in SARS-CoV S–PW5-535 but not in XBB S–PW5-535, implying that SARS-CoV S(2 P) itself is not as stable as XBB S (6 P). Fc region of PW5-535 is not visible due to its flexibility.

In XBB S–PW5-535 complex, PW5-535 binds to a hidden epitope in RBD outside the RBM, buries a surface area of 961 Å^2^ and involves a total of 17 RBD residues (Fig. 6c–e). Among these RBD residues, K386 is engaged in salt bridges with D99 and D103 of CDRH3, T385, S366, N388, Y369, K386, D389, G381, P412, D427, F429, C379, and V382 form 16 hydrogen bonds with residues from CDRs of PW5-535. Besides, Y58 of CDRH2, P95, P96, W97 of CDRL3 forms hydrophobic interaction with Y369 and P384. The R30 of CDRH1 interacts with the T531 of subdomain 1 (SD1) by the van der Waals force. SD1 is a subdomain closely related to RBD and highly conserved, which also explains that most antibodies against SD1 have a broad neutralization effect. In addition, the structural superimposition of Omicron S RBD–ACE2 (PDBID: 7WVP) onto XBB S timer–PW5-535 reveals a close contact between PW5-535 and ACE2, with only 4.8 Å between Q156 of PW5-535 light chain and E536 of ACE2 (Fig. 6f), indicating that PW5-535 binding would introduce a steric hindrance and prevent the virus from receptor recognition. This explains why PW5-535 competitively blocked the binding of ACE2 to SARS-CoV-2 S (Supplementary Fig. S6c, d).

PW5-535 induces SARS-CoV S disassembly, and buries a larger surface area of 1128 Å^2^ on SARS-CoV S monomer, involving 22 RBD residues, generating 24 pairs of hydrogen bonds and forming 2 patches of hydrophobic interactions. Compared with Omicron XBB, slight differences are observed in the interaction between PW5-535 and SARS-CoV S monomer (Fig. 6g). Firstly, K64, P102, N104 of heavy chain form extra hydrogen bonds with T359, G531, T533, L532, G368 and S370. Secondly, Y58 of heavy chain and P85, P96, and W97 of light chain do not form hydrophobic interaction but replaced by two hydrophobic patches between P102 of CDRH3 and L532, and between Y32, Y92 of CDRL and G409, F457, P440, Y408. Thirdly, S101, P102, D103 of CDRH3 formed 3 pairs of hydrogen bonds with G531, L532, T533 of SD1, further improving the affinity of the antibody. The conformational change after the unstable depolymerization of SARS-CoV S leads to exposure of more accessible epitopes.

Taken together, PW5-5 and PW5-535 both bind to a hidden epitope in S RBD outside RBM. To further explore whether there is a synergy between PW5-5 and PW5-535, various pseudotyped viruses were tested by neutralization assay. The results showed that the cocktail made of PW5-5 and PW5-535 with non-overlapping epitopes displayed a detectable synergy effect (Supplementary Fig. S11).

### Protection of PW5-5, PW5-535, and PW5-570 in golden Syrian hamster models

**Fig. 7:**
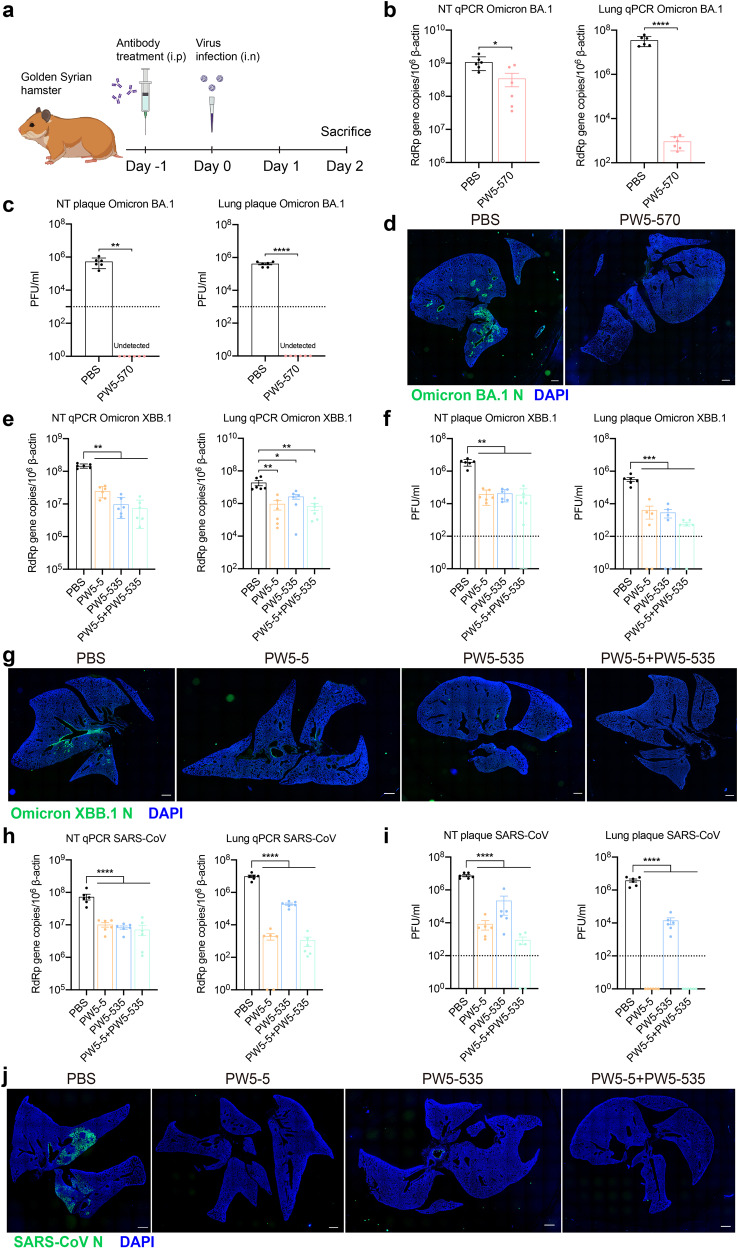
Prophylactic efficacy of PW5-5, PW5-535, and PW5-570 against SARS-CoV, SARS-CoV-2 Omicron BA.1, and XBB.1 in golden Syrian hamsters. **a** Schematic of the in vivo mAb efficacy test experiment in hamsters. **b**, **c**, **e**, **f**, **h**, **i** Viral load quantification of Omicron BA.1 (**b**, **c**), XBB.1 (**e**, **f**), and SARS-CoV (**h**, **i**) in golden hamster lung and nasal turbinate (NT) with or without treatments. Golden hamster lung and nasal turbinate samples were harvested on day 2 post SARS-CoV-2 Omicron BA.1, XBB.1, or SARS-CoV challenge and homogenized for qRT-PCR analysis and plaque assay titration (*n* = 6). **d**, **g**, **j** Immunofluorescence images of the whole section of infected hamster lungs with or without treatments. SARS-CoV-2 N protein was identified with a rabbit anti-SARS-CoV-2-N immune serum (green). Nuclei was identified with DAPI stain (blue). Scale bars in (**d**, **g**, **j**) represented 1000 μm for fourfold magnifications of the objective with tenfold magnification at the eyepiece. The experiments in (**b**, **c** and **e**, **f** and **h**, **i**) were repeated two times independently with similar results, respectively. Data represented means and SEMs from the indicated number of biological repeats. Statistical significance between groups in (**b**, **c**) was determined by unpaired *t* test and the statistical significance between groups in (**e**, **f**, **h**, **i**) was determined with one-way ANOVA. *P < 0.05, **P < 0.01, ***P < 0.001, and ****P < 0.0001.

To further evaluate the antiviral effects of the isolated neutralizing mAbs, we selected PW5-5, PW5-535, and PW5-570 for further investigation on the golden Syrian hamster model (Fig. 7a), based on the results of neutralizing assay with pseudovirus and authentic virus in vitro. Since PW5-570 demonstrated the most potent protective effects against SARS-CoV-2 variants from WT to Omicron BA.2, with IC_50_ values less than 0.01 μg/mL, it was selected to evaluate the antiviral effects against SARS-CoV-2 BA.1 in hamsters. Golden Syrian hamsters were treated with one dose of PW5-570 (20 mg/kg) or phosphate buffered saline (PBS) prophylactically through intraperitoneal injection. At 24 h post pretreatment, we intranasally inoculated the hamsters with SARS-CoV-2 BA.1, and sacrificed the hamsters at day 2 post virus inoculation. Our results suggested that prophylactic treatment of PW5-570 significantly reduced BA.1 replication in both hamster nasal turbinates and lungs, which was evidenced by the significantly lowered viral RNA-dependent RNA polymerase (RdRp) gene copy (Fig. 7b) and the undetectable infectious virus titers upon treatment (Fig. 7c). We next analyzed the expression of SARS-CoV-2 BA.1 nucleocapsid (N) protein in the harvested hamster lungs. We detected abundant viral N expression in the bronchiole epithelium as well as in the alveolar space in PBS-treated hamster lungs. In striking contrast, viral N gene expression was not detected in PW5-570-treated hamsters (Fig. 7d), suggesting the robust anti-SARS-CoV-2 potency of PW5-570.

In the meantime, we selected PW5-5 and PW5-535 to investigate their anti-sarbecovirus potential in vivo due to their broad-spectrum viral neutralizing activity against sarbecovirus. SARS-CoV-2 XBB.1 and SARS-CoV were selected to be the model sarbecoviruses for investigation. Golden Syrian hamsters were treated with one dose of PW5-5 (20 mg/kg), PW5-535 (20 mg/kg), the combination of PW5-5 and PW5-535 (10 mg/kg for each mAbs) or PBS prophylactically through intraperitoneal injection. The hamsters were inoculated with SARS-CoV-2 XBB.1 or SARS-CoV at 24 h post treatment. At day 2 post virus inoculation, the hamsters were sacrificed for sample harvest. Our results demonstrated that prophylactic treatment of PW5-5, PW5-535 or the combination of both mAbs all significantly reduced the replication of XBB.1 in the hamster nasal turbinates and lungs, with significantly lowered viral RdRp gene copy (Fig. 7e) and infectious virus titers upon treatment (Fig. 7f). In parallel, we performed immunofluorescence staining of SARS-CoV-2 N protein in the harvested hamster lungs. As shown in Fig. 7g, PW5-5, PW5-535, or PW5-5/PW5-535 dual treatment all attenuated viral N expression in the lungs of infected hamsters when compared with the PBS treatment. Importantly, prophylactic treatment of PW5-5, PW5-535 or the combination of both mAbs also significantly reduced the replication of SARS-CoV in the hamster nasal turbinates and lungs. We observed significantly lowered SARS-CoV RdRp gene copy (Fig. 7h) and infectious virus titers upon mAb treatment (Fig. 7i). Owing to the self-limiting nature of infection in the hamster model, no significant differences in body weight change were observed between the groups in any of the prophylactic settings (Supplementary Fig. S12a–c). Compared to the abundant expression of viral N protein in the PBS group of SARS-CoV infected hamster lungs, the expression of viral N protein was marginally detected in the lungs with treatment of PW5-5, PW5-535 or the combination of both mAbs (Fig. 7j), indicating that both PW5-5 and PW5-535 have the potential to neutralize SARS-CoV-2 XBB.1 and SARS-CoV in vivo.

**Fig. 8:**
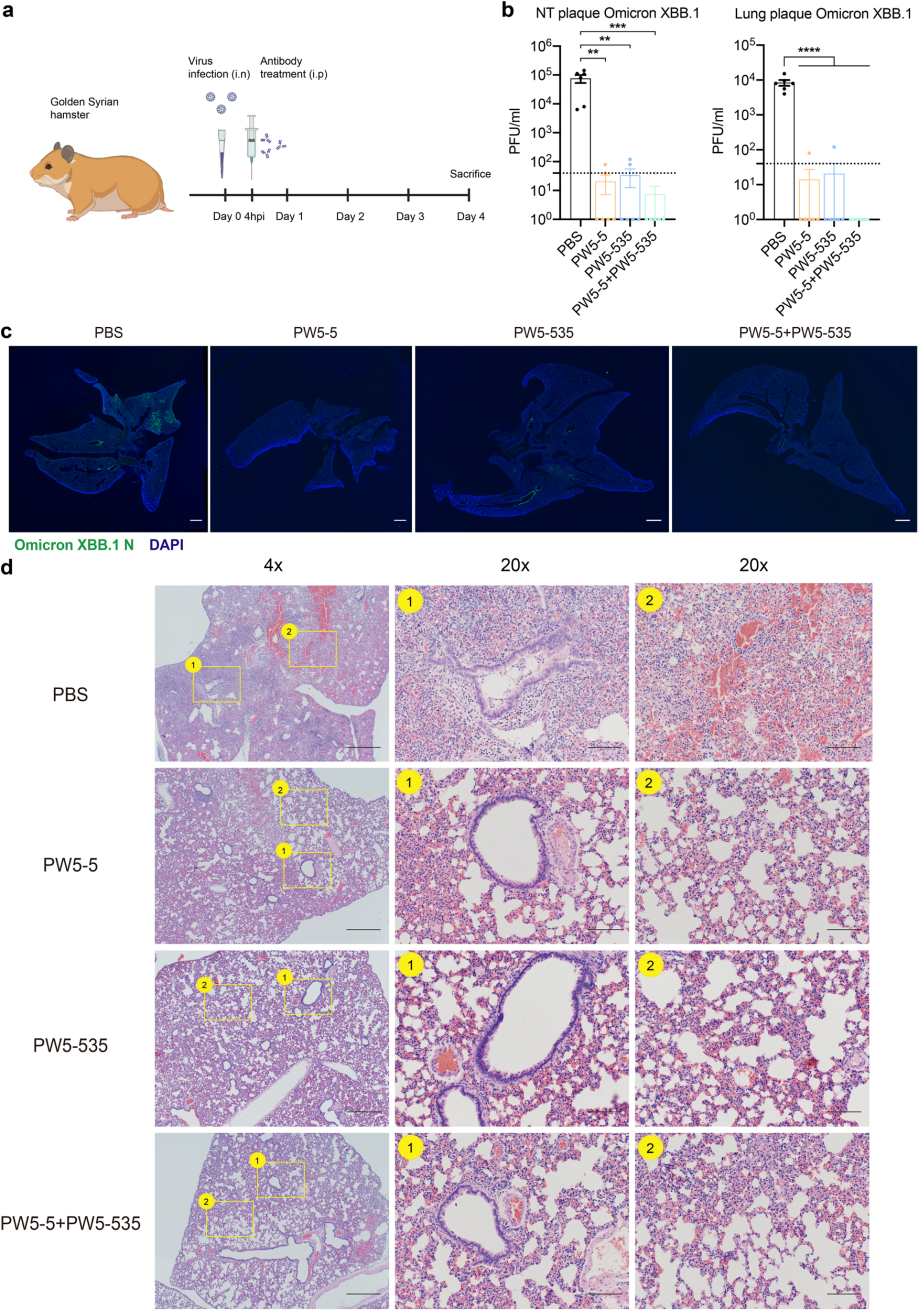
Therapeutic efficacy of PW5-5 and PW5-535 against XBB.1 in golden Syrian hamsters. **a** Schematic of the in vivo mAb efficacy test experiment in hamsters. **b** Viral load quantification of XBB.1 in golden hamster lung and nasal turbinate with or without treatments. Lung and nasal turbinate samples were harvested on day 4 post SARS-CoV-2 Omicron XBB.1 challenge and homogenized for plaque assay titration (*n* = 6). **c** Immunofluorescence images of the whole section of infected hamster lungs with or without treatments. SARS-CoV-2 N protein was identified with a rabbit anti-SARS-CoV-2-N immune serum (green). Nuclei was identified with DAPI stain (blue). Scale bars in **c** represented 1000 μm for fourfold magnifications of the objective with tenfold magnification at the eyepiece. **d** Representative H&E images of infected hamster lungs with or without treatments. A representative region of (1) bronchiole epithelium and (2) alveolar space was shown. Scale bars in **d** represented 500 or 100 μm for fourfold or 20-fold magnifications of the objective, respectively, with ×10 magnification at the eyepiece. The experiments in **b**, **d** were repeated two times independently with similar results, respectively. Data represented mean ± SEM from the indicated number of biological repeats. Statistical significance between groups in **b** was determined with one-way ANOVA. *P < 0.05, **P < 0.01, ***P < 0.001, and ****P < 0.0001.

Furthermore, we evaluated the anti-sarbecovirus potential of PW5-5 and PW5-535 in vivo with the classical therapeutic treatment approach (Fig. 8a). The golden Syrian hamsters were inoculated with SARS-CoV-2 XBB.1 intranasally. At 4 h later, the hamsters were treated with one dose of PW5-5 (20 mg/kg), PW5-535 (20 mg/kg), the combination of PW5-5 and PW5-535 (10 mg/kg for each mAbs) or PBS through intraperitoneal injection. At day 4 post virus inoculation, the hamsters were sacrificed for sample harvest. Our results demonstrated that therapeutic treatment of PW5-5, PW5-535 or the combination of both mAbs all significantly reduced the replication of XBB.1 in the hamster nasal turbinates and lungs, as evidenced by lowered infectious virus titers upon treatment (Fig. 8b). Again, we failed to observe significant difference in body weight change between these groups (Supplementary Fig. S12d). Compared to the abundant expression of viral N protein in the PBS group of XBB.1-infected hamster lungs, the expression of viral N protein was marginally detected in the lungs with the treatment of PW5-5, PW5-535 or the combination of both mAbs (Fig. 8c). Importantly, histopathological examination of the lung on day 4 post infection showed multi-focal inflammation, diffuse alveolar destruction, protein-rich fluid exudate, hyaline membrane formation, marked mononuclear cell infiltration, cell debris-filled bronchiolar lumen, and alveolar collapse in the PBS-treated group (Fig. 8d). In contrast, there was a marked attenuation of the inflammatory response in the lung of PW5-5, PW5-535 or the combination of both mAbs treated hamsters, with only mild pulmonary inflammatory infiltrates confined to the alveolar walls. Overall, our results reveal that the selected neutralizing mAbs of PW5-5 and PW5-535 have broad-spectrum antiviral effects in vivo.

## Discussion

In this study, we succeeded in creating multiple antibodies that are effective against a wide range of coronaviruses from a SARS-CoV-2 sequential vaccinated donor with cross-reactive serum-neutralizing activity. Among the 86 antibodies generated, PW5-399 and PW5-530 have efficacy against three severe human coronaviruses, although the neutralization potency is not very high. Moreover, PW5-570 displayed the most potent neutralizing activity against all SARS-CoV-2 strains prior to the Omicron BA.5 variant. Of note, PW5-4, PW5-5, and PW5-535 were found to broadly neutralize all sarbecoviruses tested. Furthermore, the binding profiles of PW5-570 demonstrated that it is a class I neutralizing antibody that blocks ACE2 binding through interaction with RBD. Distinct from some other reported mAbs of this class, such as CB6 and REGN10933, PW5-570 retains full activity against all SARS-CoV-2 strains prior to the Omicron BA.5 variant^37,38^. Sequence alignment of SARS-CoV-2 and SARS-CoV variants, combined with analysis of residues involved in PW5-570, PW5-5, and PW5-535, shows that a large proportion of PW5-570 epitope residues are mutated in the latest variants, and F486V is indeed the key mutation in the S for the antibody evasion (Supplementary Fig. S13). In contrast, PW5-5 and PW5-535 bound to more conserved epitopes hidden in RBD with non-overlapping epitopes, and most likely belong to class IV mAbs, explaining their breadth in neutralizing different variants. More importantly, PW5-5 and PW5-535 were less affected by Omicron variants, indicating that broadly neutralizing antibodies in this class can also be potential therapeutic antibodies against current VOCs and future emerging variants. In line with two class IV mAbs, GAR20 and AB-3467, PW5-535 not only competed with CR3022 but also blocked binding of ACE2 to SARS-CoV-2 S trimer^39,40^. In addition, the cocktail of PW5-5 and PW5-535 displayed detectable synergy, suggesting that using combinations of mAbs with cooperative function would both increase potency and decrease the risk of escape.

It is important to highlight that the cross-reactive antibodies in this study were identified through a combined approach of single B-cell sorting and virtual screening processes. We introduced a pipeline specifically tailored for B-cell epitope and antibody analysis. Initially, the structures of antigens and antibodies were simulated to obtain their 3D structural features. Then, the potential cross-reactive epitopes were identified by whole-protein surface traversal screening. The antigenicity scores between different mutants were contrasted by CE-BLAST, which has been previously used to screen the cross-reactive epitopes between dengue virus and zika virus^29^, as well as between SARS-CoV and SARS-CoV-2^41^. Further, for each potential cross-reactive epitope, the ranking of the antibody was obtained by scoring the binding ability based on the micro-environment matching of physic-chemical properties between the epitope regions and CDR regions. Our methodology successfully pinpointed 86 antibodies that showcased potential cross-reactive capabilities. Further experiments showed that a majority of these antibodies exhibited commendable binding ability to three different coronaviruses, and some of them even displayed potent neutralizing activity (Fig. 2). This underscores the proficiency of our in silico approach in discerning broad-spectrum antibodies based on structural information.

Structural characterization demonstrated that PW5-570 occupied part of the RBM region like other class I antibodies to prevent receptor attachment, similar to CB6, B38, and C102, and the binding region is a concave plane at the top of RBD, which is highly coexisting with the RBM region^31,42,43^. The binding region of hACE2 and RBD region is directly blocked by steric hindrance, which is an effective and common neutralizing antibody type, but it lost its neutralizing ability against many Omicron VOCs, as RBM is the region with the most variation of all the variants. In addition, the formation of trimer dimer indicates potential steric hindrance or virion aggregation to neutralize virions. However, it is worth noting that broad-spectrum antibodies targeting more conserved epitopes typically have much lower neutralizing activity than antibodies targeting RBM regions.

Despite the relatively high sequence conservation of SARS-CoV and SARS-CoV-2, there are differences in antigenicity, and antibodies that can broadly neutralize SARS-CoV-2 VOCs and SARS-CoV are rarely reported. In this study, PW5-5 and PW5-535 had high neutralizing activities against SARS-CoV-2 VOCs and SARS-CoV. As shown in Supplementary Fig. S14, the binding epitope of PW5-5 are highly overlapped with FD20 and S2H97, hidden in the inner side of highly conserved RBD, including the key epitopes R355, R357, K462, and P463 covered by both FD20 and S2H97, as well as the key residue E465 involved in the formation of three hydrogen bonds in FD20 epitopes^44,45^. Compared with S2H97 (IC_50_: 2046 ng/mL), PW5-5 has significantly enhanced neutralizing activity against SARS-CoV (IC_50_: 10 ng/mL), increasing by ~200 times. PW5-5 strengthened the binding force between the antibody and S protein through a large number of hydrogen bonds and hydrophobic interactions, and the binding epitopes were narrow, so it could better cope with mutations. Sequence alignment between SARS-CoV and SARS-CoV-2 in the RBD region showed that PW5-5 epitopes were highly conserved, with 11 of the 13 residues in contact with SARS-CoV-2 being identical (Supplementary Fig. S13), and the high similarity between the two RBDs was consistent with PW5-5’s cross-reactivity in binding and neutralizing SARS-CoV. Structural analysis showed that the binding of PW5-5 induced RBD to shift outward and enter the “extra wide open” state, resulting in conformational instability of RBD. This phenomenon is similar to n3130v inducing S trimer to form a “wide-up” state, but the deflection angle is larger, causing S trimer to be more unstable^46^. This conclusion is consistent with our observation that incubation with PW5-5 and Omicron XBB S trimers leads to the gradual decomposition of some trimers into monomers. The binding epitope of PW5-535 is similar to the class IV antibody AB-3467, which is located in the hidden surface and around residues such as Y369, F377, K378, Y380, P384, etc (Supplementary Fig. S14)^40^. Both of them can effectively neutralize SARS-CoV-2 and SARS-CoV through their light chains, which block the ACE2–RBD interaction by introducing steric hindrance. The binding epitope of PW5-535 overlaps partially with that of CR3022^34^. The difference is that CR3022 cannot bind SARS-CoV-2 with P384 mutation, but Y58 of PW5-535 CDRH2 can form hydrogen bond with P384. At the same time, Y58 of CDRH2, P95, P96, and W97 of CDRL3 formed hydrophobic interactions with Y369 and P384, which endowed PW5-535 with neutralization effect on SARS-CoV and SARS-CoV-2. The binding area of PW5-535 is larger and extends down to SD1, and the SD1 sequence is highly conserved among SARS-CoV-2 variants. This epitope may explain the broad-spectrum neutralizing activity of PW5-535. In summary, we analyzed the different binding epitopes and neutralizing mechanisms of PW5-570, PW5-5, and PW5-535 through cryo-EM. The epitopes of PW5-5 and PW5-535 are highly conserved and can be paired with other non-competitive neutralizing antibodies, indicating potential pairing strategies for cocktail therapy against SARS-CoV-2 and novel emerging coronaviruses.

Apart from the in vitro neutralization activity and structural characterization, we also demonstrated that prophylactic treatment with a single dose of PW5-570 significantly protected golden Syrian hamsters from infection by the Omicron BA.1 variant, indicating that PW5-570 might represent a best-in-class anti-SARS-CoV-2 antibody for use in the prophylactic setting. More importantly, we demonstrated that PW5-5 and PW5-535 exhibited excellent in vivo prophylactic protection against both the circulating SARS-CoV-2 XBB.1 variant and SARS-CoV in the hamster model, indicating that PW5-5 and PW5-535 have the potential to be developed as pan-sarbecoviruses antivirals for immunotherapy and transmission prevention clinically. To this end, future vaccine design should consider stabilizing the binding interface of the immunogen for interaction with PW5-5 and PW5-535.

There are several limitations in this study to be considered when interpreting the results. To understand the frequency of PW5-5- and PW5-535-like broadly neutralizing mAbs among sequential vaccinees, we need to investigate other responders who show equally potent broadly neutralizing antibody responses. Furthermore, we only tested a single dose of PW5-5, PW5-535 or PW5-570 for prophylactic efficacy in the hamster model. However, these mAbs should be tested for therapeutic efficacy in the hamster model as well to provide useful information in support of clinical development of similar broadly neutralizing antibodies. Besides, the Fc-mediated effector functions of these mAbs have not been evaluated. In addition, we did not isolate and characterize mAbs recognizing other regions of S, including NTD and S2.

## Materials and methods

### Protein expression and purification

The constructs used for expression of stabilized soluble MERS-CoV, SARS-CoV, SARS-CoV-2, and Omicron BA.1 S2P S trimer proteins were obtained from our previous studies^47^. The mammalian expression plasmid for dimeric soluble ACE2 was purchased from Addgene. SARS-CoV-2 NTD (aa 1–305) and RBD (aa 319–541) were separately cloned into mammalian expression vector pCMV3 with an 8× His tag and 2× Strep-tag II tags at the C terminus. Expi293 cells were used for transient transfection with the suitable S2P-stabilized S-expression plasmids or other vectors by using 1 mg/mL polyethylenimine (PEI, Polysciences). The supernatant was harvested and the S trimer was purified using Ni-NTA resin from Smart Lifesciences (Changzhou, China, Ni Smart Beads 6FF: SA036100) in accordance with the manufacturer’s protocol five days after transfection. Prior to use, all proteins were further evaluated for size and purity through SDS-PAGE.

### Sorting for S trimer-specific B cells and single-cell B-cell receptor sequencing

Flow cytometry of PBMCs from one healthy donor with five times of vaccinations were conducted following methods described previously^47^. All collections were conducted according to the guidelines of the Declaration of Helsinki and approved by the Institutional Review Board of the Ethics Committee of Huashan Hospital (2021-749). This participant provided written informed consent. Briefly, PBMCs were stained with LIVE/DEAD Fixable Yellow Dead Cell Stain Kit (Invitrogen) at ambient temperature for 20 min, followed by washing with RPMI-1640 complete medium and incubation with 10 μg/mL SARS-CoV-2 Omicron BA.1- and MERS-CoV-S trimers with His tag at 4 °C for 1 h. Afterward, the cells were washed again and incubated with a cocktail of flow cytometry antibodies, containing CD3 APC-H7 (BD Biosciences), CD19 BV421 (BD Biosciences), CD27 APC (BD Biosciences), and anti-His PE (Biolegend), at 4 °C for 1 h. Stained cells were then washed, resuspended in RPMI-1640 complete medium and sorted for S trimer-specific memory B cells (CD3^−^CD19^+^CD27^+^S trimer^+^ live single lymphocytes). The sorted cells were loaded into the BD Rhapsody single-cell analysis platform, which could effectively capture and separate single cells. The BCR library preparation and quality control were performed according to the manufacturer’s protocol and sequenced on the NovaSeq PE150 platform (Illumina).

### In silico screening of cross-reactive antibodies

To detect the potential cross-reactive antibodies for SARS-CoV-2 WT, SARS-CoV-2 Omicron, and MERS-CoV among all 684 antibodies obtained from BCR sequencing, an in silico pipeline was constructed. It is an integrated computational pipeline that could provide in silico screening of broad-reactive antibodies for a group of antigen mutants, which includes five steps: (1) obtaining the 3D structures of antigen protein based on four different types of template, (2) mapping the epitope regions on the modeled S protein, including the real crystalized epitopes and simulated epitopes, (3) selecting the broad-spectrum epitope regions by calculating the antigenicity similarity score of each epitope regions across the three viruses through CE-BLAST, (4) generating the antibody structure library by modeling the 3D structures for all 684 antibodies, (5) calculating the physicochemical features and structure features for structures from the potential broad-spectrum epitope library and the antibody structure library to perform virtual screening between epitope and antibody. Top-ranking antibodies through the in silico screening were selected for further validation. Detailed information on each step can be found below.

### Structure modeling

The 3D structures of S proteins of SARS-CoV-2 WT, Omicron and MERS-CoV were constructed through SWISS-MODEL^48^. Considering the S monomer in the trimer structures contains two different modes, up and down, four different trimer structures include: (1) three RBD up (3-RBD-up), (2) two RBD up and one RBD down (2-RBD-up), (3) one RBD up and two RBD down (1-RBD-up), and (4) three RBD down (3-RBD-down). Structures of four trimer types are illustrated in Supplementary Fig. S3. The templates of the above four trimer types are illustrated in Supplementary Table S1. Antibody structures were modeled through ABodyBuilder^49^, a fully automated Ab structure prediction server, to generate modeled structures of all 684 paired antibodies in our dataset.

### Epitope mapping

After structure modeling, the epitopes regions were mapped on the S trimers which involve crystalized real epitopes and simulated virtual epitopes. For crystalized real epitope, we derived 1349 SARS-CoV-2 S–antibody complexes from SabDab^49^. After removing redundancy (according to sequence similarity of 100%), 1083 S–antibody complexes which involve 1083 real epitope regions were remained for further analysis. For virtual epitope simulation, we first calculated the accessible surface area (ASA) for each residue in the S protein based on the template structures through Pymol^50^. Then, the surface residues were defined as those residues with ASA over 1 Å^2^. Typically, for each surface residue$r$, all residues in the S protein within 10 Å were defined as the epitope residues of surface patch ${P}_{r}$. Here, we generated 10,909 epitopes for both SARS-CoV-2 WT and Omicron S, and 9608 virtual epitopes for MERS-CoV S.

### Cross-reactive epitope prediction

For any real or virtual epitope ${{EP}}_{n}$, the cross-protective score between SARS-CoV-2 ${WT}-{{EP}}_{n}$, ${Omicron}-{{EP}}_{n}$, and ${MERS}-{{EP}}_{n}$ were calculated through CE-BLAST^29^. The potential cross-reactive epitopes were defined as those epitope regions that obtain cross-reactive scores above the threshold across all three viruses. By setting the threshold of 0.7 for CE-BLAST score, 132 potential cross-reactive epitopes were detected, including 4 real epitopes and 128 virtual epitopes. After mapping the potential cross-reactive epitope on the templates, each epitope region contains three epitope files (SARS-CoV-2 WT, SARS-CoV-2 Omicron, and MERS-CoV).

### Antibody screening

For each potential cross-reactive epitope, we screened all the 684 antibodies through the patch model of special epitope prediction of protein antigens tool-for monoclonal antibody (SEPPA-mAb)^30^. Typically, the epitope patch of the cross-reactive epitope and the CDR patch of the antibody were encoded into a group of fingerprints to calculate the binding score between each epitope patch and CDR patch. Then, for each epitope patch, the score of CDR patch against MERS-CoV, SARS-CoV-2 WT, and SARS-CoV-2 Omicron were ranked among all 684 antibodies. If one CDR patch can be ranked within the top 5 (<1%) among all 684 mAbs in all three epitope files for one epitope region, this antibody will be considered as the potential cross-reactive antibody for the specific epitope region. Then, 80 antibodies obtained by screening, along with 6 high-frequency antibodies, were selected for further experimental test (Supplementary Table S2).

### Antibody expression and purification

Monoclonal antibodies tested in this study were constructed and produced at Fudan University. For each antibody, variable genes were codon optimized for human cell expression and synthesized by HuaGeneTM (Shanghai, China) into plasmids (gWiz) that encode the constant region of human IgG1 heavy or light chain. Antibodies were expressed in Expi293F (Thermo Fisher, A14527) by co-transfection of heavy and light chain expressing plasmids using polyethylenimine (Polyscience) and cells were cultured at 37 °C with shaking at 125 rpm and 8% CO_2_. Supernatants were also collected on day 5 for antibody purification using MabSelectTM PrismA (Cytiva, 17549801) affinity chromatography.

### Production of pseudoviruses

Plasmids encoding the MERS-CoV, SARS-CoV, SARS-CoV-2, and SARS-CoV-2 variants spikes, as well as the spikes with single or combined mutations were synthesized. Expi293F cells were grown to 3 × 10^6^/mL before transfection with the indicated spike gene using Polyethylenimine. Cells were cultured overnight at 37 °C with 8% CO_2_ and VSV-G pseudotyped DG-luciferase (G*DG-luciferase, Kerafast) was used to infect the cells in DMEM at a multiplicity of infection of 5 for 4 h before washing the cells with PBS/2% fetal bovine serum (FBS) three times. The next day, the transfection supernatant was collected and clarified by centrifugation at 300× *g* for 10 min. Each viral stock was then incubated with 20% I1 hybridoma (anti-VSV-G; ATCC, CRL-2700) supernatant for 1 h at 37 °C to neutralize the contaminating VSV-G pseudotyped DG-luciferase virus before measuring titers and making aliquots to be stored at –80 °C.

### Pseudovirus neutralization

Neutralization assays were performed by incubating pseudoviruses with serial dilutions of monoclonal antibodies or sera, and scored by the reduction in luciferase gene expression as described previously^51^. In brief, Vero-E6 cells were seeded in a 96-well plate at a concentration of 2 × 10^4^ cells per well. On the following day, pseudoviruses were incubated with serial dilutions of the test samples in triplicate for 30 min at 37 °C. The mixture was added to cultured cells and incubated for an additional 24 h. The luminescence was measured by the Luciferase Assay System (Beyotime). IC_50_ was defined as the dilution at which the relative light units were reduced by 50% compared with the virus control wells (virus + cells) after subtraction of the background in the control groups with cells only. The IC_50_ values were calculated using nonlinear regression in GraphPad Prism.

### Viruses and biosafety

SARS-CoV GZ50 (GenBank accession number AY304495) was an archived clinical isolate at the Department of Microbiology, HKU. SARS-CoV-2 WT D614G (GISAID: EPL_ISL_497840), B.1.1.7/Alpha (GenBank: OM212469), B.1.351/Beta (GenBank: OM212470), B.1.617.2/Delta (GenBank: OM212471), Omicron BA.1 (EPI_ISL_6841980), Omicron BA.2 (GISAID: EPI_ISL_9845731), Omicron BA.5 (GISAID: EPI_ISL_13777658), Omicron XBB.1 (GISAID: EPI_ISL_15602393) and Omicron BQ.1.1 (GISAID: EPI_ISL_16342297) strains were isolated from the respiratory tract specimens of laboratory-confirmed COVID-19 patients in Hong Kong^52,53^. SARS-CoV-2 was cultured using Vero-E6-TMPRSS2. SARS-CoV was cultured in Vero-E6 cells. All the viruses were titrated by plaque assays. All experiments with infectious SARS-CoV-2 and SARS-CoV were performed according to the approved standard operating procedures of the Biosafety Level 3 facility at the Department of Microbiology, HKU.

### Authentic SARS-CoV and SARS-CoV-2 variants neutralization

An end-point dilution assay in a 96-well plate format was performed to measure the neutralization activity of select purified mAbs as described previously^47^. In brief, each antibody was serially diluted (fivefold dilutions) starting at 100 μg/mL. Triplicates of each mAb dilution were incubated with indicated live virus at a MOI of 0.1 in DMEM with 7.5% inactivated fetal calf serum for 1 h at 37 °C. After incubation, the virus–antibody mixture was transferred onto a monolayer of Vero-E6 cells grown overnight. The cells were incubated with the mixture for 70 h. The cytopathic effects were visually scored for each well in a blinded fashion by two independent observers. The results were then converted into percentage neutralization at a given mAb concentration, and means ± SEM were plotted using a five-parameter dose–response curve in GraphPad Prism 8.0.

### Epitope mapping by ELISA

For the epitope binding ELISA, 50 ng per well of indicated S trimer, 50 ng per well of RBD, and 100 ng per well of NTD were coated onto ELISA plates at 4 °C overnight. The ELISA plates were then blocked with 300 μL blocking buffer (0.5% BSA and 5% Skim Milk) in PBST (0.05% Tween-20 in PBS) at 37 °C for 2 h. Afterwards, purified antibodies were serially diluted using dilution buffer (0.2% BSA and 2% Skim Milk in PBST), and incubated at 37 °C for 1 h. Next, 100 μL of 5000-fold diluted Peroxidase AffiniPure goat anti-human IgG (H + L) antibody (Promega) was added into each well and incubated for 1 h at 37 °C. The plates were washed between each step with PBST three times. Finally, the TMB substrate (Promega) was added and incubated before the reaction was stopped using 1 M sulfuric acid. Absorbance was measured at 450 nm.

For the competition ELISA, the first antibody at the concentration of 2 μg/mL was coated on ELISA plates and incubated at 4 °C overnight. The ELISA plates were then blocked with 300 μL blocking buffer (0.5% BSA and 5% Skim Milk) in PBST at 37 °C for 2 h. After washing, SARS-CoV-2 S trimer with 2× Strep-tag II tags was diluted to a final concentration of 2 μg/mL, followed by mixing with 50 μg/mL of the second competition antibodies or PBS blank control. After incubation at 37 °C for 1 h, plates were washed three times with PBST, and the 5000-fold diluted anti-Strep-Tactin HRP conjugate antibody (IBA Life Science) was subsequently added. Then, the plates were incubated at 37 °C for 1 h again. The plates were then washed and developed with TMB, and absorbance was read at 450 nm after the reaction was stopped. The competitive percentage of two antibodies was calculated with reference to the PBS blank control.

For the ACE2 competition ELISA, 100 ng of soluble ACE2 protein was immobilized on the plates at 4 °C overnight. The unbound ACE2 was washed away by PBST and then the plates were blocked. After washing, 100 ng of SARS-CoV-2 S trimer with 2× Strep-tag II tags in 50 μL dilution buffer was added into each well, followed by addition of another 50 μL of serially diluted competitor antibodies and then incubation at 37 °C for 1 h. The ELISA plates were washed three times with PBST and then 100 μL of 5000-fold diluted anti-Strep-Tactin HRP conjugate antibody (IBA Life Science) was added into each well for another 1 h at 37 °C. The plates were then washed and developed with TMB, and absorbance was read at 450 nm after the reaction was stopped.

### Cryo-EM data collection and processing

The indicated S trimer at 5 mg/mL was mixed with PW5-5, PW5-535 or PW5-570 at 5 mg/mL in a 1:1.2 molar ratio (S trimer/IgG), incubated at 4 °C for 1 h, and the indicated S trimer–antibody complexes were separated on a Superose 6 Increase 10/300 GL and diluted to 0.7 mg/mL in 20 mM Tris, pH 8.0, 200 mM NaCl. XBB/SARS-CoV S–PW5-535 complexes gradually depolymerize after incubation and then be diluted directly after incubation for freezing samples. A 3 μL complex sample was added to a freshly glow-discharged holey amorphous nickel-titanium alloy film supported by 400-mesh gold grids. Vitrobot IV (FEI, Thermo Fisher Scientific) was used to immerse the sample in liquid ethane and frozen for 2-s, –3blot force, and 10-s wait time.

Cryo-EM data were collected on a TITAN Krios G4 Transmission electron microscopy (Thermo Fisher Scientific) operated at 300 kV, equipped with a Falcon 4i and a Selectris X Imaging filter (Thermo Fisher Scientific) setting to a slit width of 20 eV. EPU software at 300 kV in AFIS mode was used for automated data collection. EER movie stack was collected in super-resolution mode, at a nominal magnification of 105,000× for PW5-535 and PW5-570, and 130,000× for PW5-5, corresponding to a physical pixel size of 1.19 Å and 0.932 Å, dose fractioned to 1737 frames and 1080 frames, respectively. Defocus ranges from –1.0 μm to –3.0 μm, with a total dose of about 50 e^–^/Å^2^.

All the data processing was performed using either modules on, or through, RELION v3.1 or cryoSPARC v4.2.1. The movie stacks were binned 2 × 2, dose-weighted, and motion-corrected using MotionCorr2 within RELION. Then CTF was estimated using Gctf. All micrographs were imported to cryoSPARC for patched CTF-estimation, particle picking and 2D classification. Good particles from selected good micrographs were imported to Relion and then 3D refinement and 3D classification were performed. The reported resolutions above were based on the gold-standard Fourier shell correlation (FSC) 0.143 criterion. All the visualization of maps and handedness correction are performed in UCSF Chimera. Sharpened maps are generated using DeepEMhancer. The resolution was estimated according to the gold-standard Fourier shell correlation (FSC) 0.143 criterion. All the data processing procedures are summarized in Supplementary Figs. S15–19. The maps were sharpened by DeepEMhancer, and handedness was corrected using UCSF Chimera.

### Model building and refinement

SWISS-MODEL and Omicron S–FD01 (PDB:7WOW), SARS-CoV S–CV38-142 (PDBID:7LM9), Omicron BA.1 S–MO1 Fab (PDBID:8H3M) were used for generating initial model. Models were fitted into the maps using UCSF Chimera, and further manually adjusted in COOT, followed by several rounds of real space refinement in PHENIX. Model validation was performed using PHENIX. Figures were prepared using UCSF Chimera and UCSF ChimeraX. The statistics of data collection and model refinement are listed in Supplementary Table S3.

### Hamster protection experiment

All experiments in the study complied with the relevant ethical regulations for research. The use of animals followed all relevant ethical regulations and was approved by the Committee on the Use of Live Animals in Teaching and Research of The University of Hong Kong. Male and female golden Syrian hamsters, aged 4–6 weeks old, were obtained from the HKU Centre for Comparative Medicine Research (CCMR). To evaluate the antiviral effects of selected antibodies, hamsters were pre-treated intraperitoneally with one dose of PW5-570 (20 mg/kg), PW5-5 (20 mg/kg), PW5-535 (20 mg/kg), or combination of PW5-5 (10 mg/kg) and PW5-535 (10 mg/kg), respectively, one day before virus inoculation or 4 h post virus inoculation. In brief, the hamsters treated with PW5-570 or the vehicles were inoculated with 1 × 10^4^ PFU SARS-CoV-2 BA.1 pre-diluted in 50 μL PBS intranasally, 24 h after antibody pretreatment. Meanwhile, the hamster treated with PW5-5, PW5-535, combination of PW5-5 and PW5-535, or the vehicles were inoculated with 5 × 10^3^ PFU SARS-CoV or 2 × 10^4^ PFU SARS-CoV-2 XBB.1 pre-diluted in 50 μL PBS intranasally, respectively. All intranasal treatment in hamsters was performed under intraperitoneal ketamine (100 mg/kg) and xylazine (10 mg/kg) anesthesia. The health status and body weight of hamsters were monitored on a daily basis until the animal was sacrificed or euthanized because of reaching the humane endpoint of the experiment. Hamsters were sacrificed on day 2 or day 4 post infection. Nasal turbinate (NT) tissues and lung tissues were harvested for immunofluorescence staining, hematoxylin and eosin (H&E) staining, qRT-PCR, or plaque assays as we previously described^54,55^.

### Immunofluorescence staining

Infected hamster lungs were fixed for 24 h in 10% formalin. The fixed samples were washed with 70% ethanol and embedded in paraffin by TP1020 Leica semi-enclosed benchtop tissue processor (Leica Biosystems, Buffalo Grove, IL, USA) and sectioned with microtome (Thermo Fisher Scientific). Sectioned samples were dewaxed by serially diluted xylene, ethanol, and double-distilled water in sequence. To retrieve the antigens, the sectioned samples were boiled with antigen unmasking solution (H-3300, Vector Laboratories) at 85 °C for 90 s, followed by Sudan black B and 1% BSA blocking for 30 min, respectively. The in-house rabbit anti-SARS-CoV-2-N immune serum or in-house rabbit anti-SARS-CoV immune serum was used as the primary antibodies to stain the N protein of SARS-CoV-2 or SARS-CoV accordingly by incubation at 4 °C overnight. The secondary antibody, goat anti-rabbit IgG (H + L) cross-adsorbed secondary antibody (A-11008), was purchased from Thermo Fisher Scientific. The anti-fade mounting medium with DAPI (H-1200, Vector Laboratories, Burlingame, CA, USA) was used for mounting and DAPI staining. Images were acquired by using Nikon Ti2-E Widefield Microscope (Japan, Tokyo).

### Plaque assays

Vero-E6-TMPRSS2 cells were seeded in 12-well plates at day 0. The harvested supernatant samples were serially diluted by tenfold and inoculated to the cells for 2 h at 37 °C. After inoculation, the cells were washed with PBS three times, and covered with 2% agarose/PBS mixed with DMEM/2% FBS at 1:1 ratio. The cells were fixed with 4% paraformaldehyde after 72 h incubation. Fixed samples were stained with 0.5% crystal violet in 25% ethanol/distilled water for plaque visualization.

### Supplementary information

Supplementary Information
