# Supplementary material for: Potent and broadly neutralizing antibodies against sarbecoviruses induced by sequential COVID-19 vaccination
Source: Cell Discov. 2024 Feb 6;10:14. doi: 10.1038/s41421-024-00648-1 (PMC10847457; doi:10.1038/s41421-024-00648-1)
Supplement: Supplementary file 1 — Supplementary Information [file 41421_2024_648_MOESM1_ESM.pdf]

Supplemental Information

Potent and broadly neutralizing antibodies against sarbecoviruses induced by sequential COVID-19 vaccination

Xiaoyu Zhao[1,#], Tianyi Qiu[2,#], Xiner Huang[3,#], Qiyu Mao[1,4,#], Yajie Wang[1,4,#], Rui Qiao[1,#], Jiayan Li[1], Tiantian Mao[5,6], Yuan Wang[6], Yewei Cun[5], Caicui Wang[6], Cuiting Luo[3], Chaemin Yoon[3], Xun Wang[1], Chen Li[1], Yuchen Cui[1], Chaoyue Zhao[1], Minghui Li[1], Yanjia Chen[1], Guonan Cai[1], Wenye Geng[7], Zixin Hu[1,8], Jinglei Cao[9], Wenhong Zhang[9], Zhiwei Cao[5,*], Hin Chu[3,*], Lei Sun[1,4,*], Pengfei Wang[1,*]

This file includes:

Supplementary Figures S1-19

Supplementary Tables S1-3

Supplementary Fig. S1

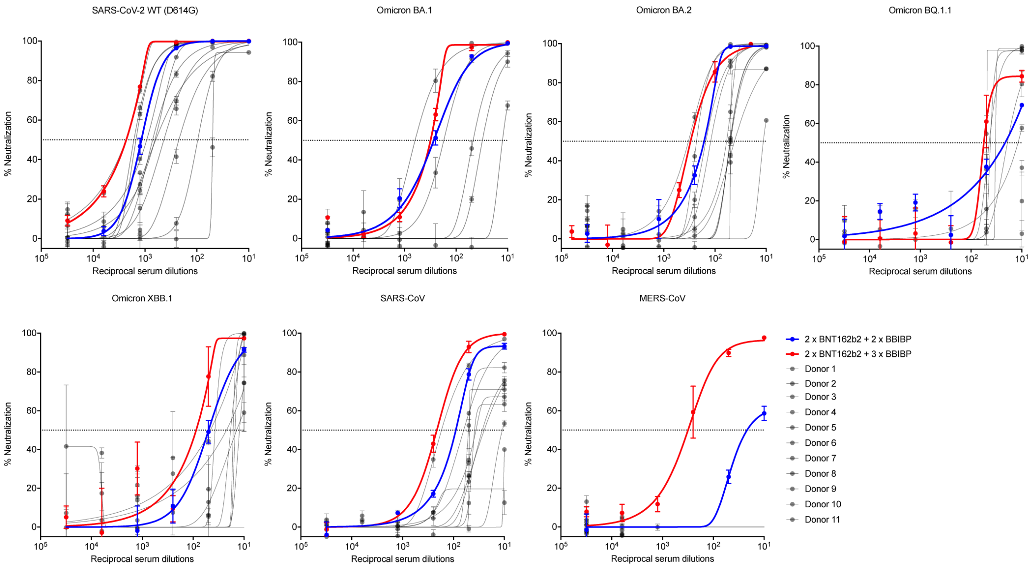

Supplementary Fig. S1 Neutralization curves for sera collected from a healthy volunteer after the fourth- (2 x BNT162b2 + 2 x BBIBP-CorV) or the fifth-vaccination dose (2 x BNT162b2 + 3 x BBIBP-CorV), or from individuals vaccinated with three-dose BBIBP-CorV only (*n* = 11) against indicated coronavirus. The data are representative of one of at least three independent experiments and are presented as the mean ± SEM.

Supplementary Fig. S2

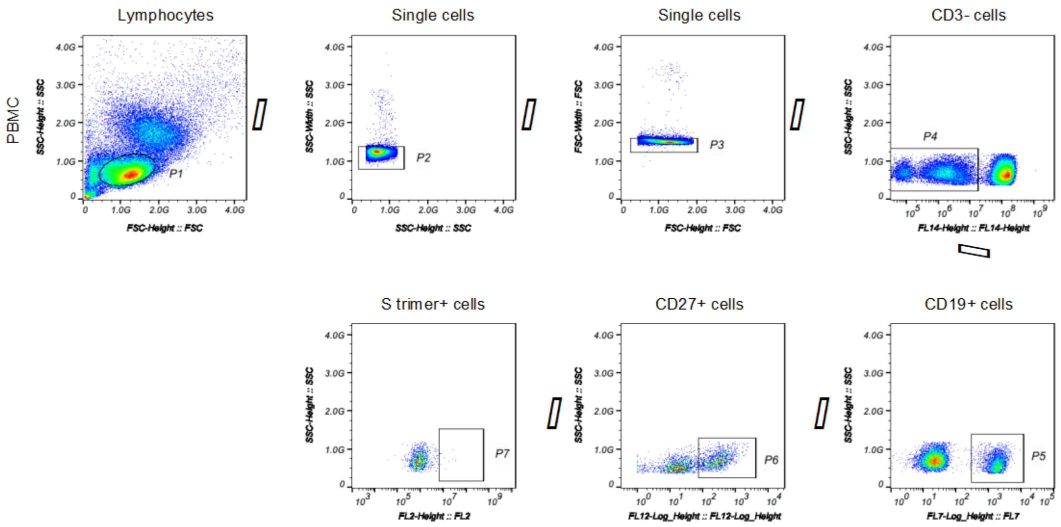

Supplementary Fig. S2 Sorting strategy for the isolation of MERS-CoV or SARS-CoV-2 Omicron S trimer–specific memory B cells using flow cytometry. The sorting primarily focused on live memory B lymphocytes that were $CD3^-$, $CD19^+$, and $CD27^+$. The final step focused on those cells that bind to the MERS-CoV or SARS-CoV-2 Omicron S trimer.

Supplementary Fig. S3

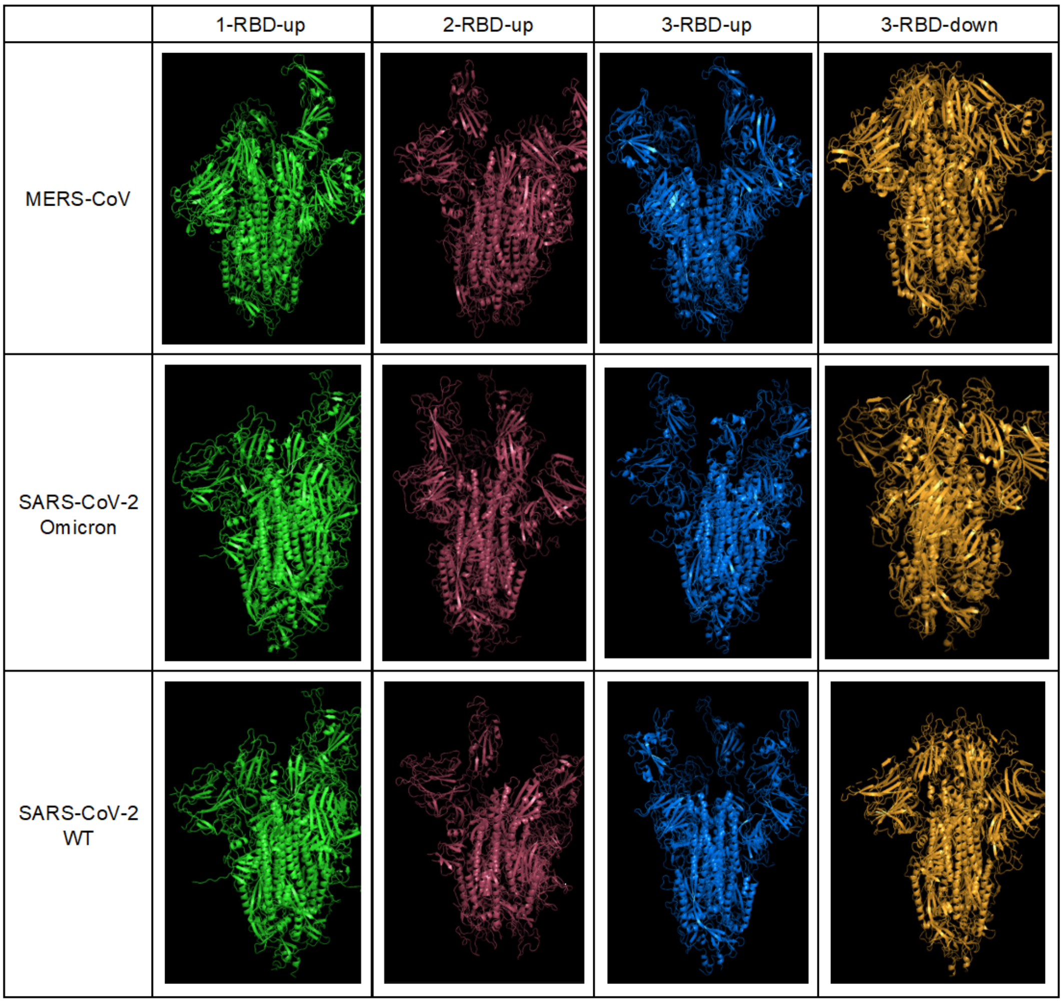

Supplementary Fig. S3 Structure illustration of four S trimer types of SARS-CoV-2 WT, Omicron and MERS-CoV.

Supplementary Fig. S4

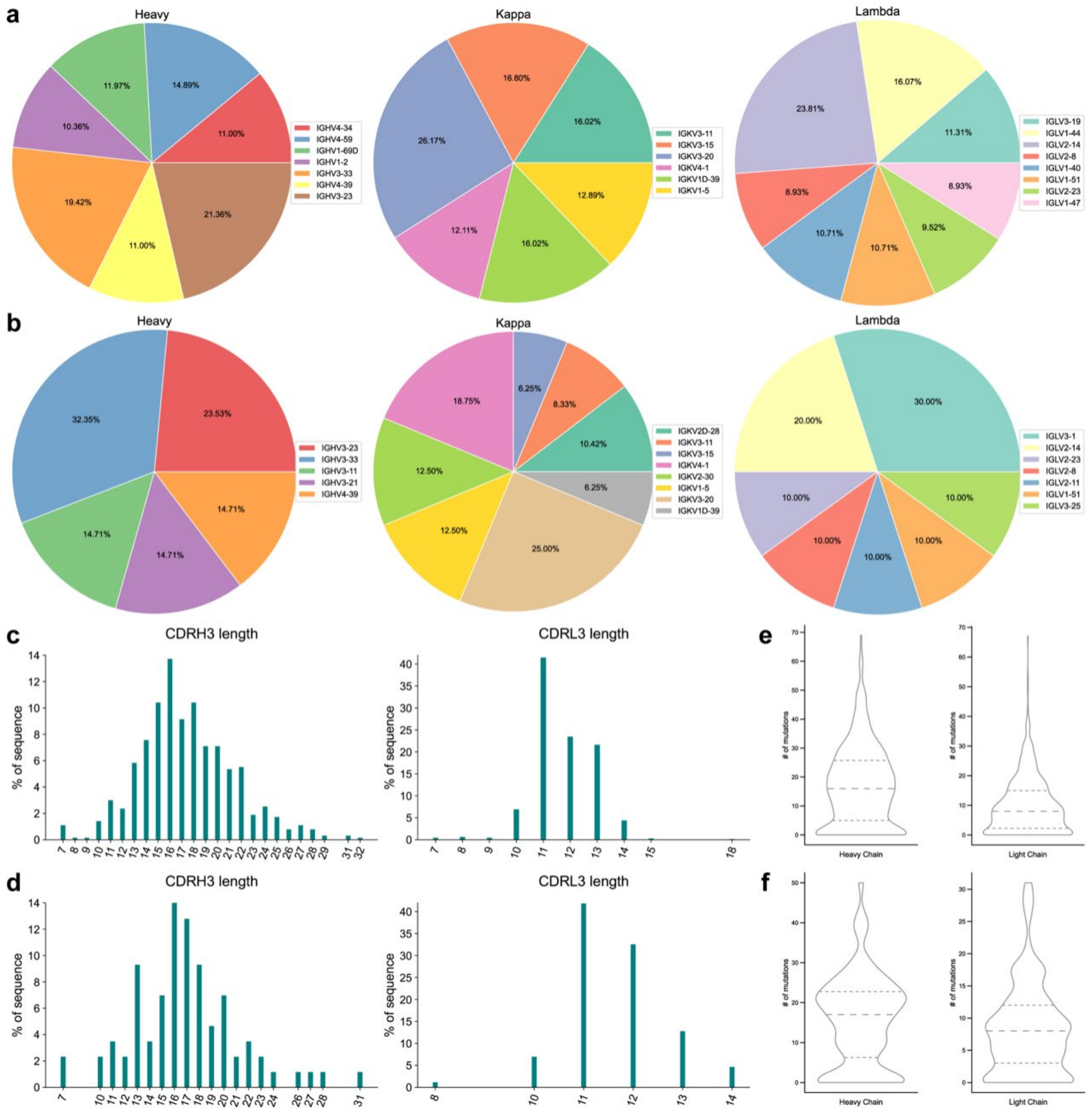

Supplementary Fig. S4 The V gene usage was assigned for paired heavy, kappa and lambda chains recovered from all the 684 sorted S trimer-specific mAbs (**a**) and the 86 mAbs further selected by bioinformatic analysis (**b**). Percentages are shown on graph for V chains that make up more than 5% of the total for each sort. The CDR3 length distribution for the heavy and light chains of the 684 mAbs (**c**) and the 86 mAbs (**d**). The number of nucleotide mutations in heavy and light chains of the 684 mAbs (**e**) and the 86 mAbs (**f**).

Supplementary Fig. S5

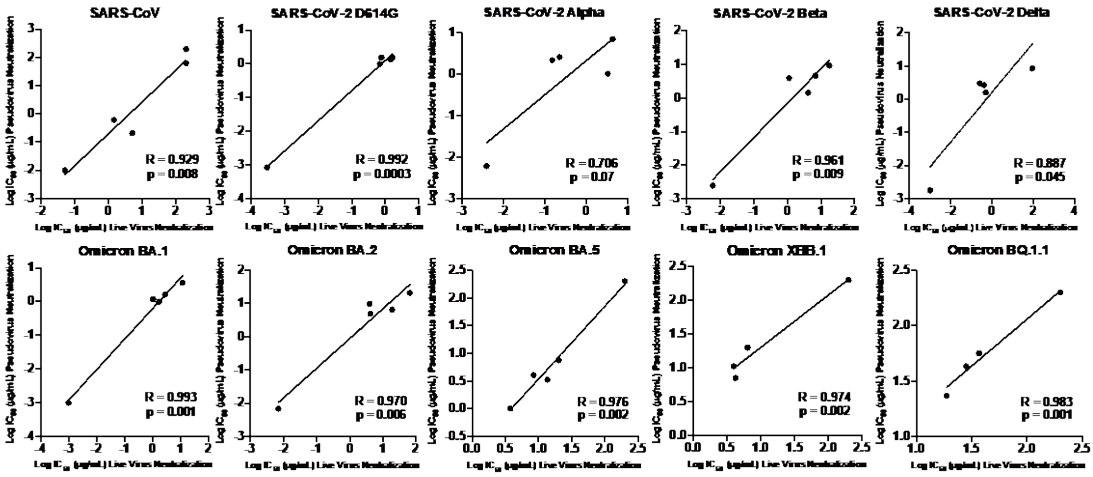

Supplementary Fig. S5 Correlation of live virus neutralization titers versus pseudovirus neutralization titers against SARS-CoV, SARS-CoV-2 and SARS-CoV-2 variants. The Pearson correlation coefficient (R) and the *P* value were calculated using GraphPad Prism. Experiments were performed in triplicates for all mAbs tested.

Supplementary Fig. S6

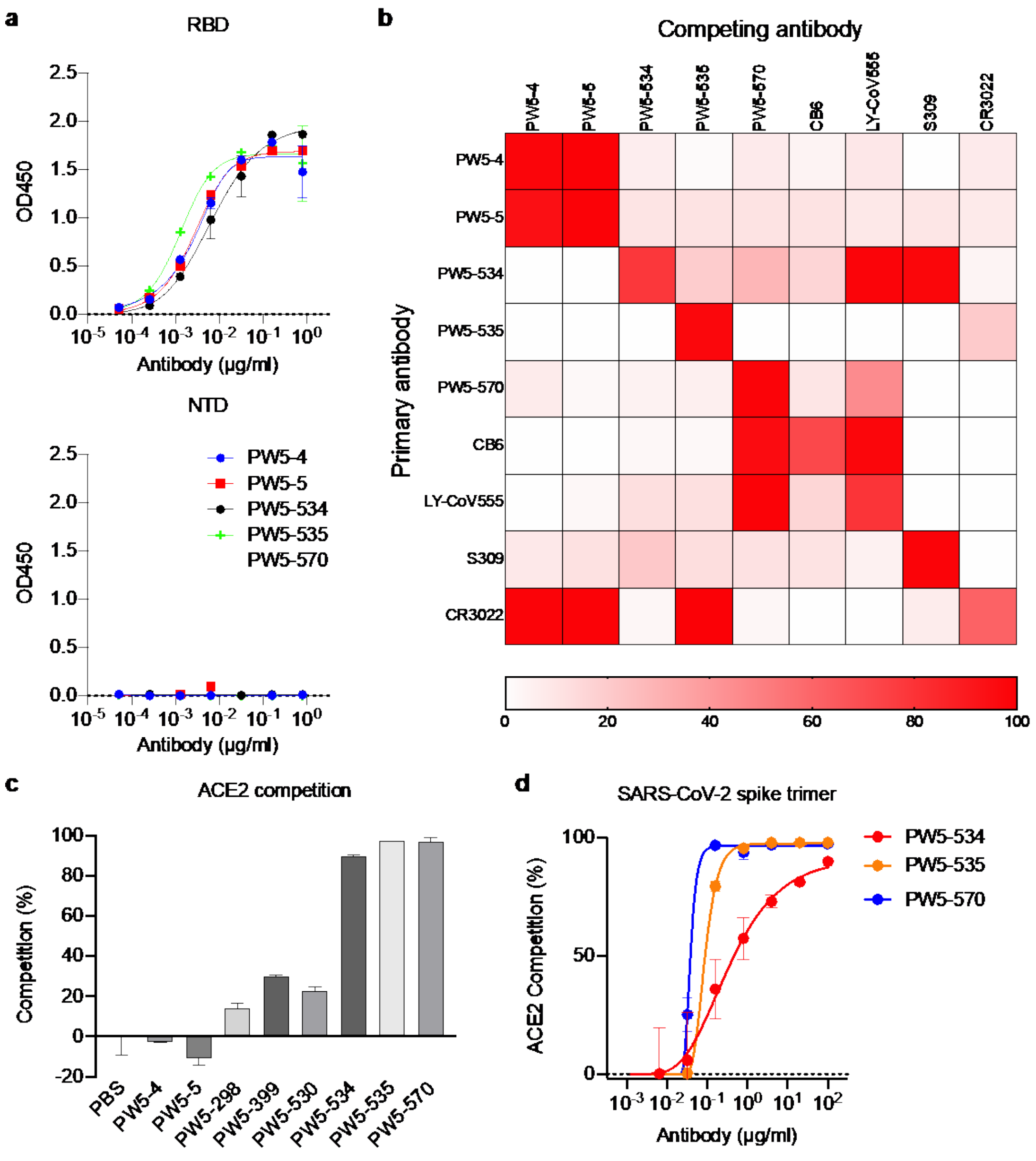

Supplementary Fig. S6 Binding properties of the selected mAbs. a Binding profiles of 5 purified potent neutralizing mAbs against the RBD (up), and NTD (down) of SARS-CoV-2. **b** Epitope mapping of the selected mAbs using a competition ELISA. A heatmap was used to show the competition percentages between two antibodies, as compared with CB6, LY-CoV555, S309 and CR3022, whose epitopes have been well defined. **c** Antibodies competitively blocked the ACE2 binding to SARS-CoV-2 S trimer as measured by ELISA. Recombinant human ACE2 protein and phosphate buffer

solution (PBS) was used as controls. **d** PW5-534, PW5-535, and PW5-570 blocked the ACE2 binding to SARS-CoV-2 S-trimer in a dose dependent manner. The data are representative of one of at least three independent experiments and are presented as the mean ± SD.

Supplementary Fig. S7

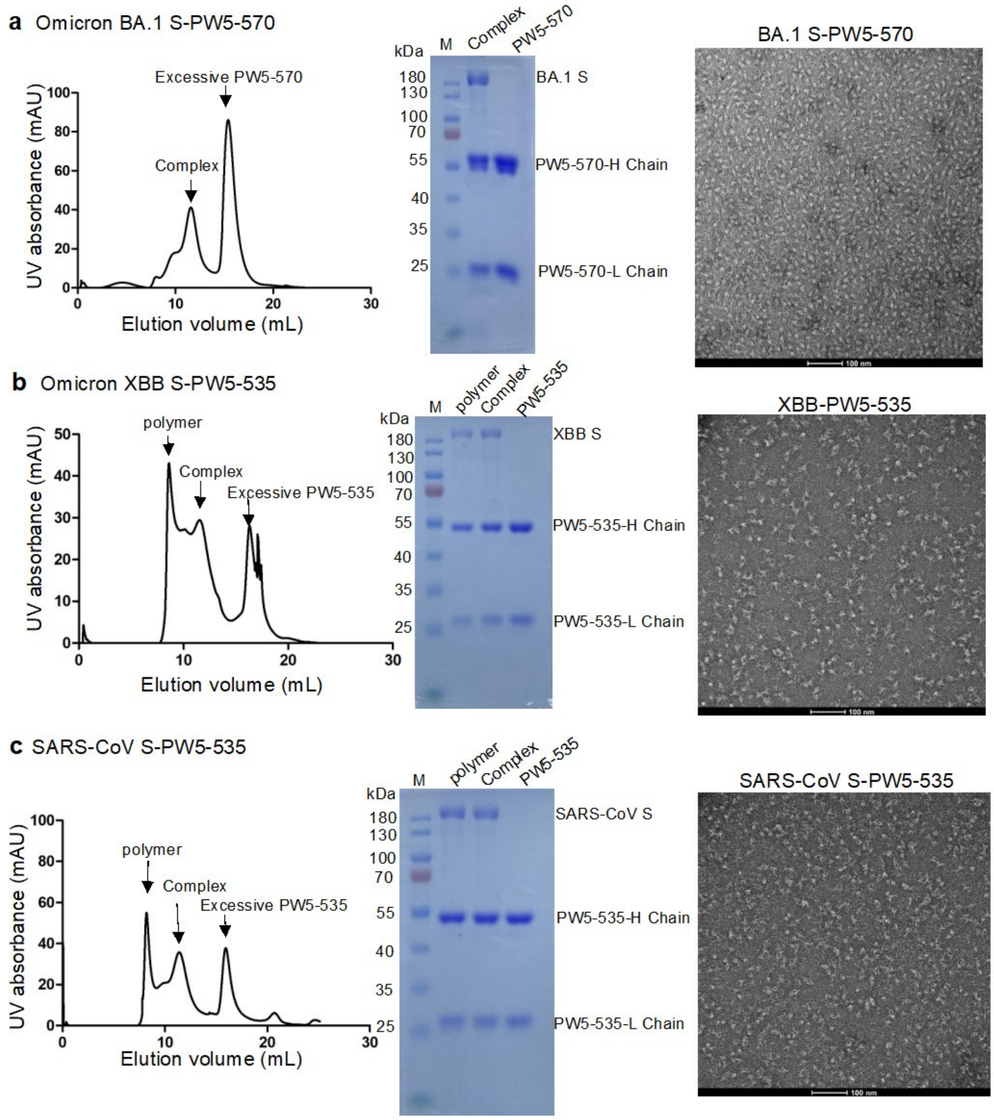

Supplementary Fig. S7 Sample purification of BA.1 S-PW5-570, XBB S-PW5-535 and SARS-CoV S-PW5-535 complex. **a** Purification and negative stain images of BA.1 S-PW5-570 complex. **b**, **c** Purification and negative stain images of XBB S-PW5-535 complex and SARS-CoV S-PW5-535 complex.

Supplementary Fig. S8

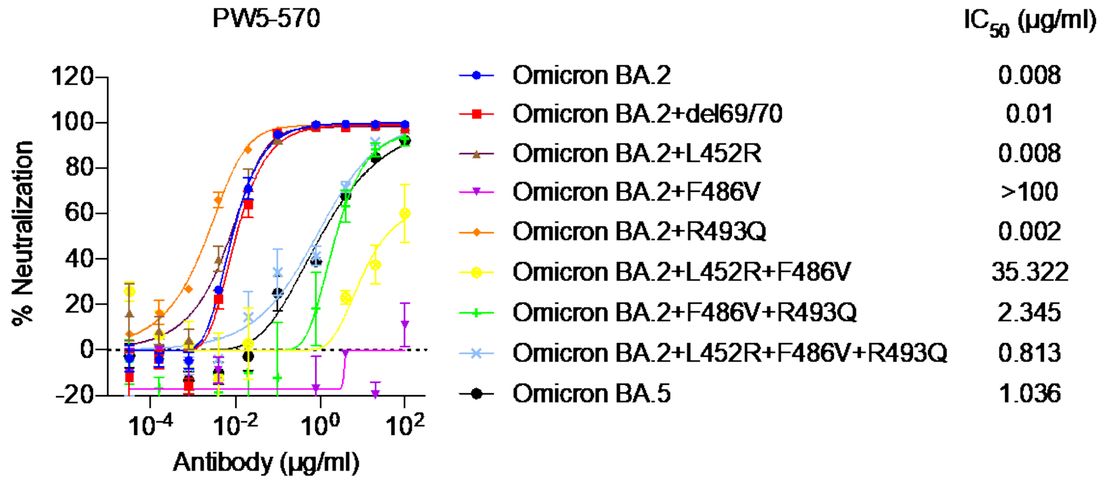

Supplementary Fig. S8 Key mutations conferring PW5-570 antibody resistance. Neutralization of Omicron BA.2 and Omicron BA.5, as well as the Omicron BA.2-based VSVs with single or combined mutations by PW5-570. The data are representative of one of at least three independent experiments and are presented as the mean ± SEM.

Supplementary Fig. S9

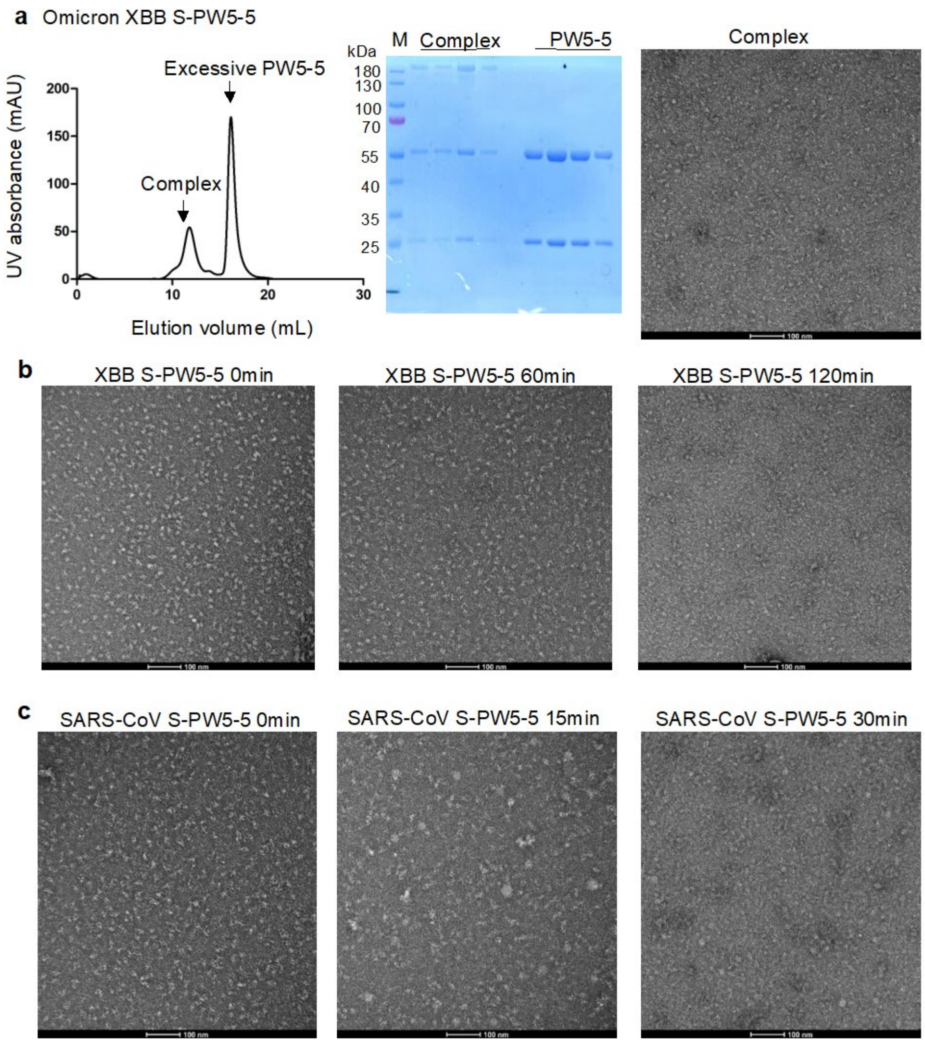

Supplementary Fig. S9 Sample purification of XBB S-PW5-5 and SARS-CoV S-PW5-5 complex. a Purification of XBB S-PW5-5 complex. The gel-filtration curve showed that PW5-5 and S protein can form complex. **b**, **c** Negative stain images of XBB S-PW5-5 complex and SARS-CoV S-PW5-5 complex, showing that incubating S with PW5-5 induced S trimer disassembling.

Supplementary Fig. S10

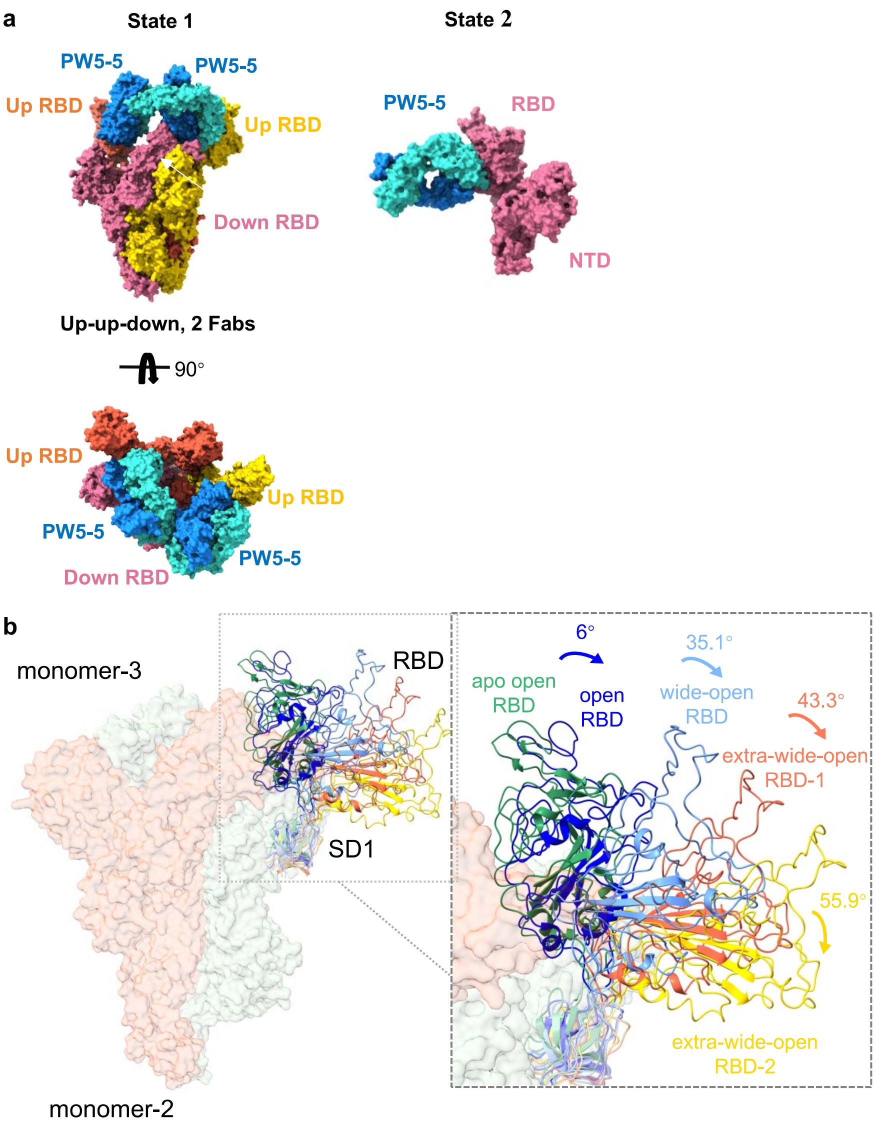

Supplementary Fig. S10 Cryo-EM structures of the SARS-CoV S in complex with antibody PW5-5 (**a**) and PW5-5 induce obvious movement of RBD (**b**). Green: apo state up RBD (PDBID: 7WVN). Dark blue: up RBD bound with bispecific antibody FD01 (PDBID: 7WOQ). Light blue: up RBD bound with nanobody bn03 (7WHK). Orange: up-RBD 1 in 3-up state XBB S bound with PW5-5. Gold: up-RBD 2 in 3-up state XBB S bound with PW5-5.

Supplementary Fig. S11

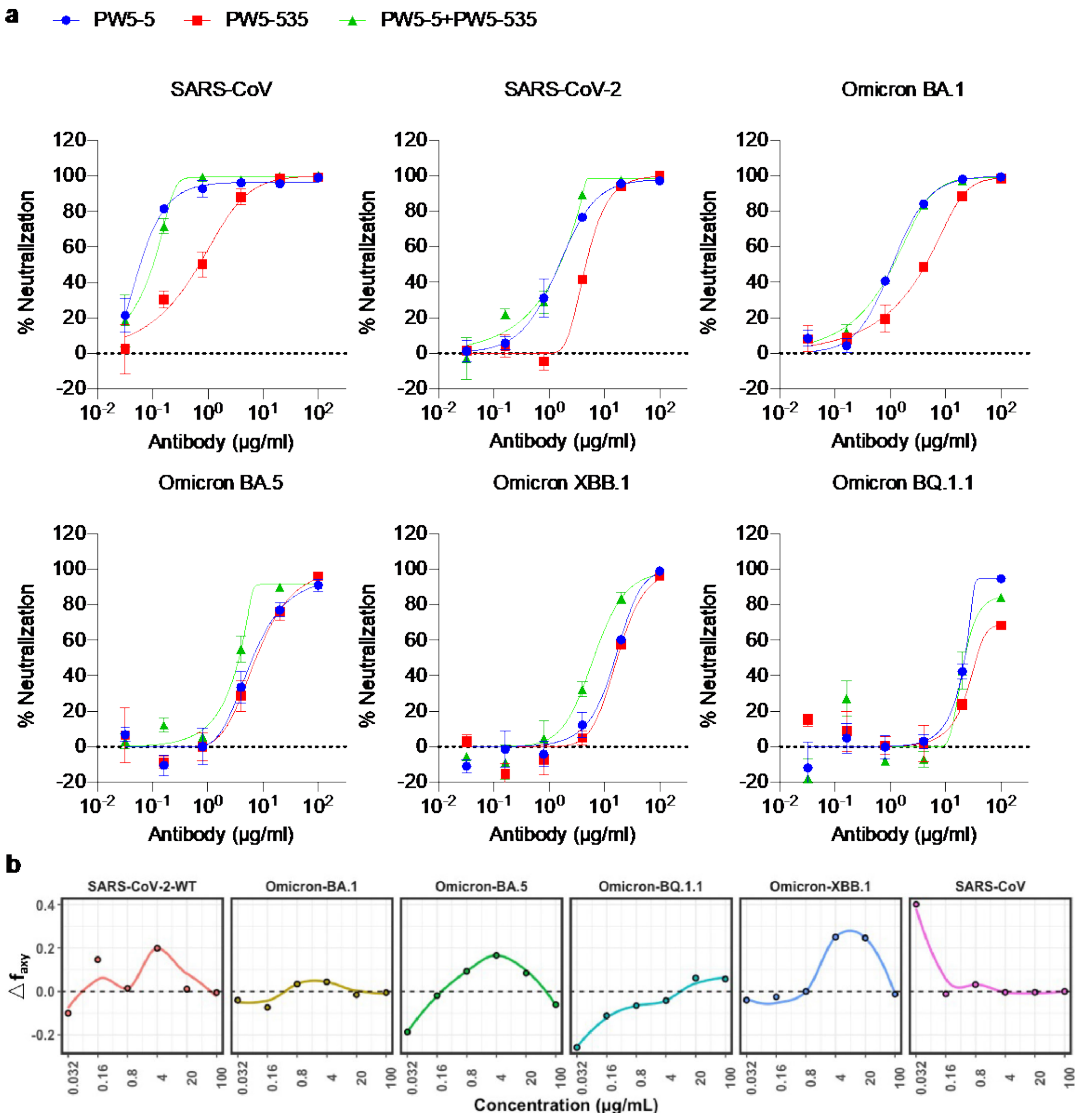

Supplementary Fig. S11 Neutralization synergy effect by PW5-5 and PW5-535. a Neutralization of SARS-CoV, SARS-CoV-2, and current Omicron subvariants by PW5-5, PW5-535 and their combination, respectively. **b** The Bliss independence model was utilized for calculation of synergy for PW5-5 and PW5-535 combinations. Synergy is defined as $\Delta f_{axy} > 0$, while $\Delta f_{axy} < 0$ indicates antagonism. The data are representative of one of at least three independent experiments and are presented as the mean ± SEM.

Supplementary Fig. S12

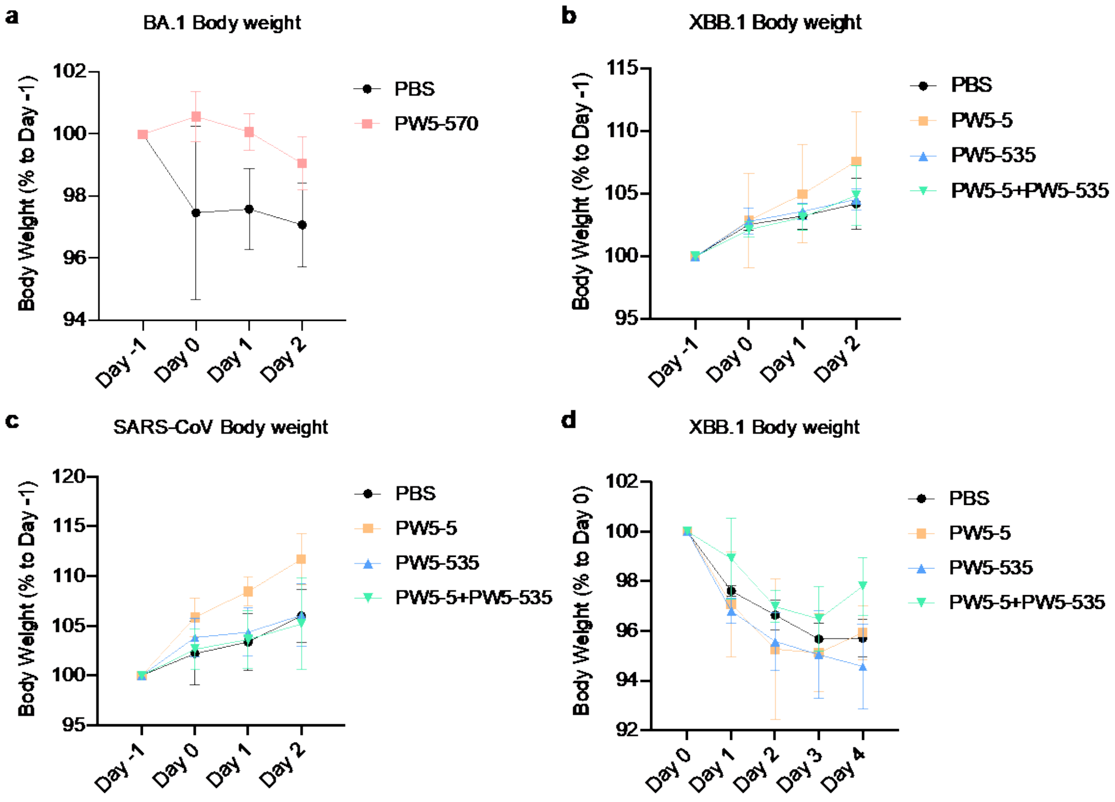

Supplementary Fig. S12 The body weight change of golden Syrian hamsters infected with SARS-CoV-2 BA.1 (**a**), XBB.1 (**b**) or SARS-CoV (**c**) with prophylactic treatment of the indicated antibody. **d** Body weight changes of therapeutic treatment of PW5-5 and PW5-535 against SARS-CoV-2 XBB.1.

Supplementary Fig. S13

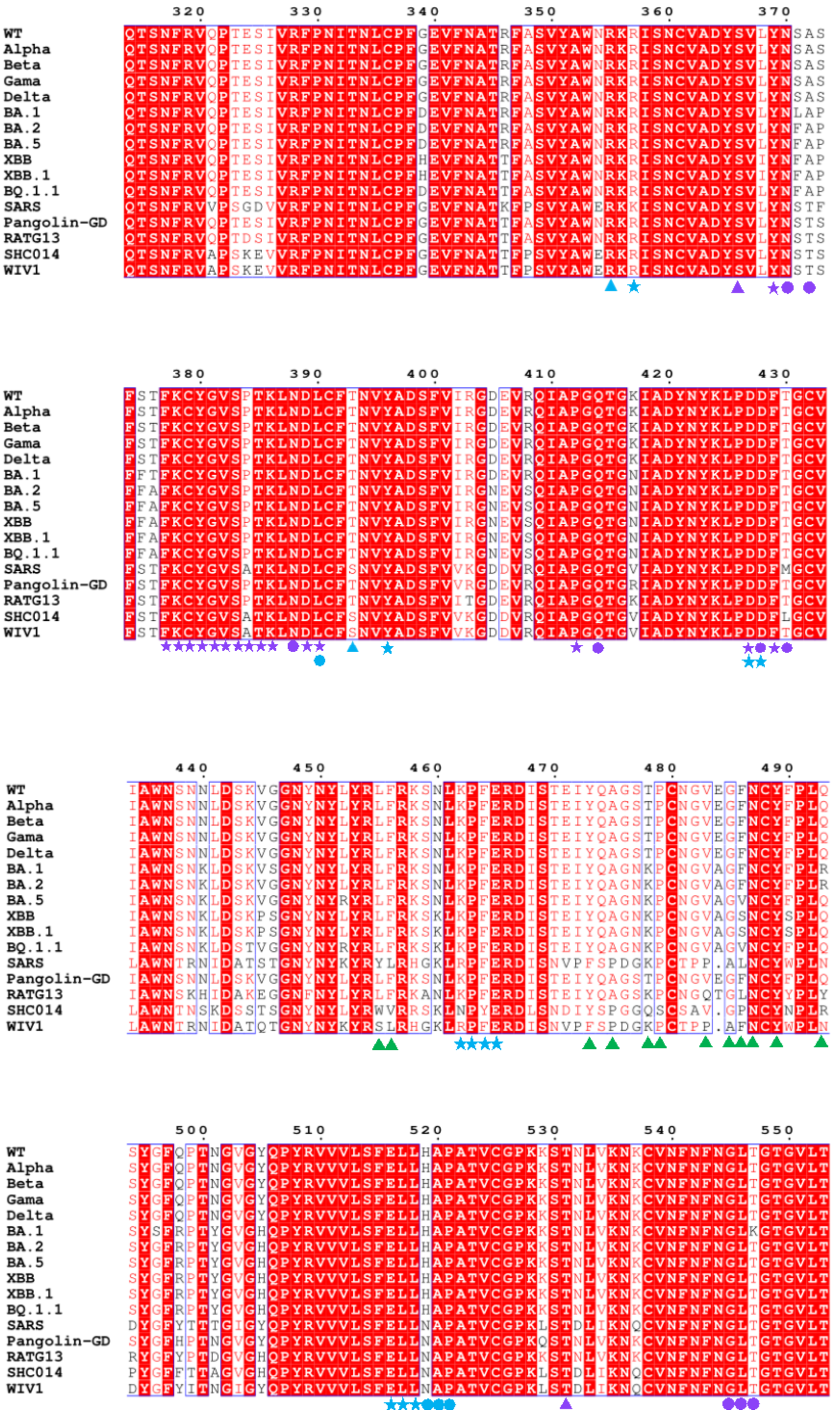

Supplementary Fig. S13 S RBD sequence alignment of SARS-CoV-2 variants, SARS-CoV and other sarbecoviruses. Conserved amino acids are highlighted as red. Residues involved in PW5-570, PW5-5 and PW5-535 binding are marked in green, blue and purple, respectively. Residues involved only in XBB S binding are marked in triangles, only in SARS-CoV S binding are marked in circles and both in XBB and SARS-CoV S binding are marked in stars.

Supplementary Fig. S14

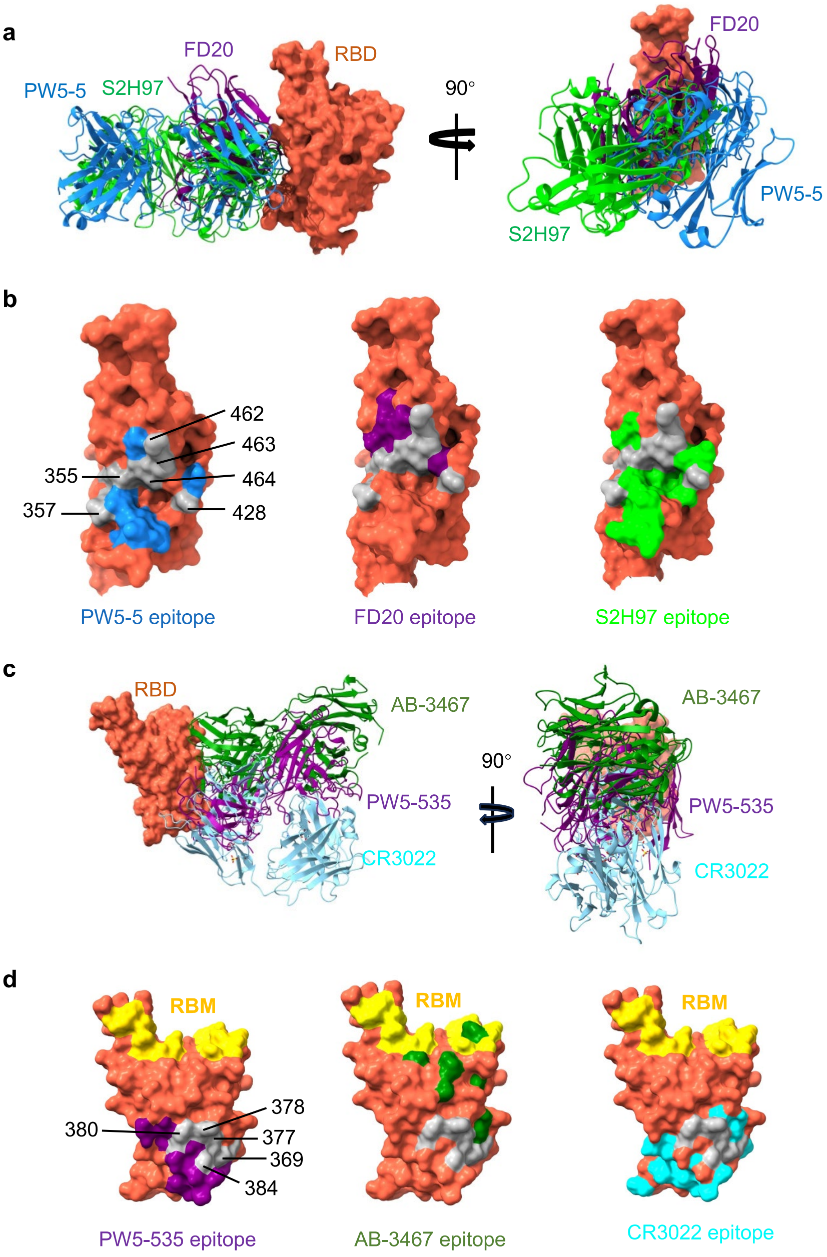

Supplementary Fig. S14 Antibodies targeting similar epitopes on the SARS-CoV-2 S RBD with PW5-5 and PW5-535. a Comparison of PW5-5 (blue), FD20 (PDBID:7CYV) (purple) and S2H97 (PDBID:7M7W) (green) upon binding to RBD. **b** Surface representation of RBD showing the buried regions by PW5-5, FD20 and S2H97, respectively. **c** Comparison of PW5-535 (purple), AB-3467 (PDBID:7MSQ) (green), CR3022 (PDBID: 6W41) (cyan) and ACE2 upon binding to RBD. **d** Surface representation of RBD showing the buried regions by PW5-535, AB-3467 and CR3022, respectively.

Supplementary Fig. S15

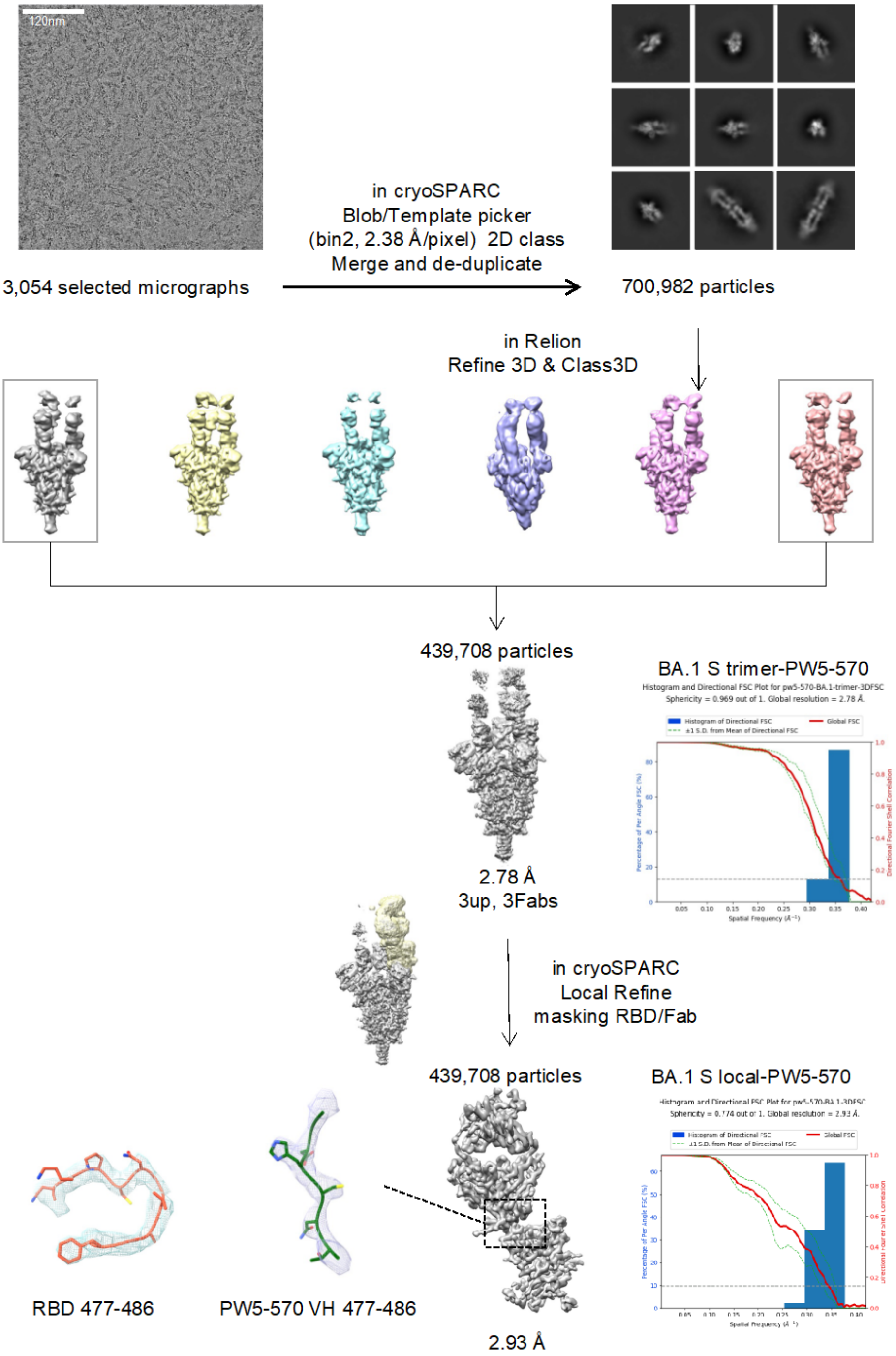

Supplementary Fig. S15 Data processing flowchart of BA.1-S-PW5-570 complex.

Supplementary Fig. S16

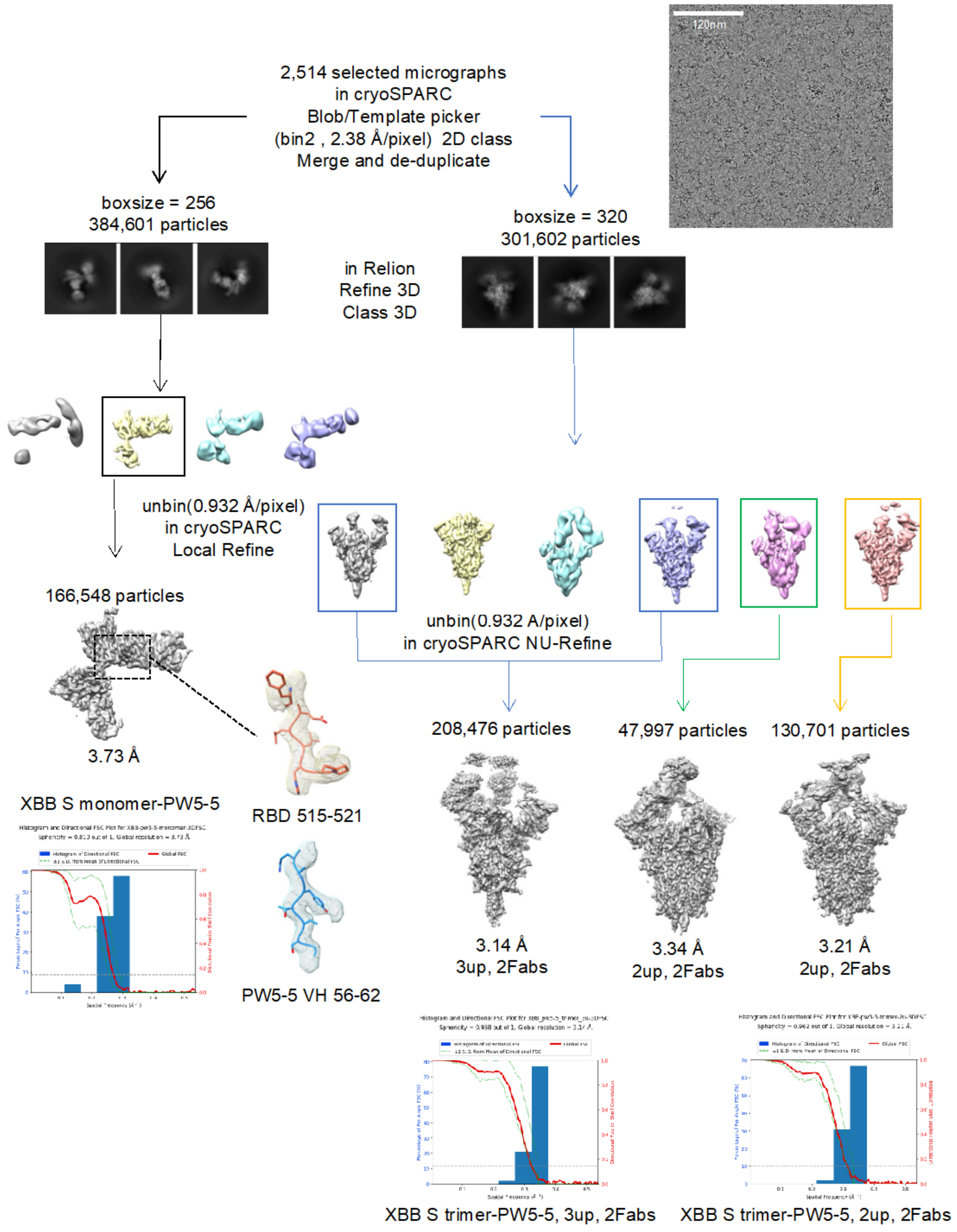

Supplementary Fig. S16 Data processing flowchart of XBB-S-PW5-5 complex.

Supplementary Fig. S17

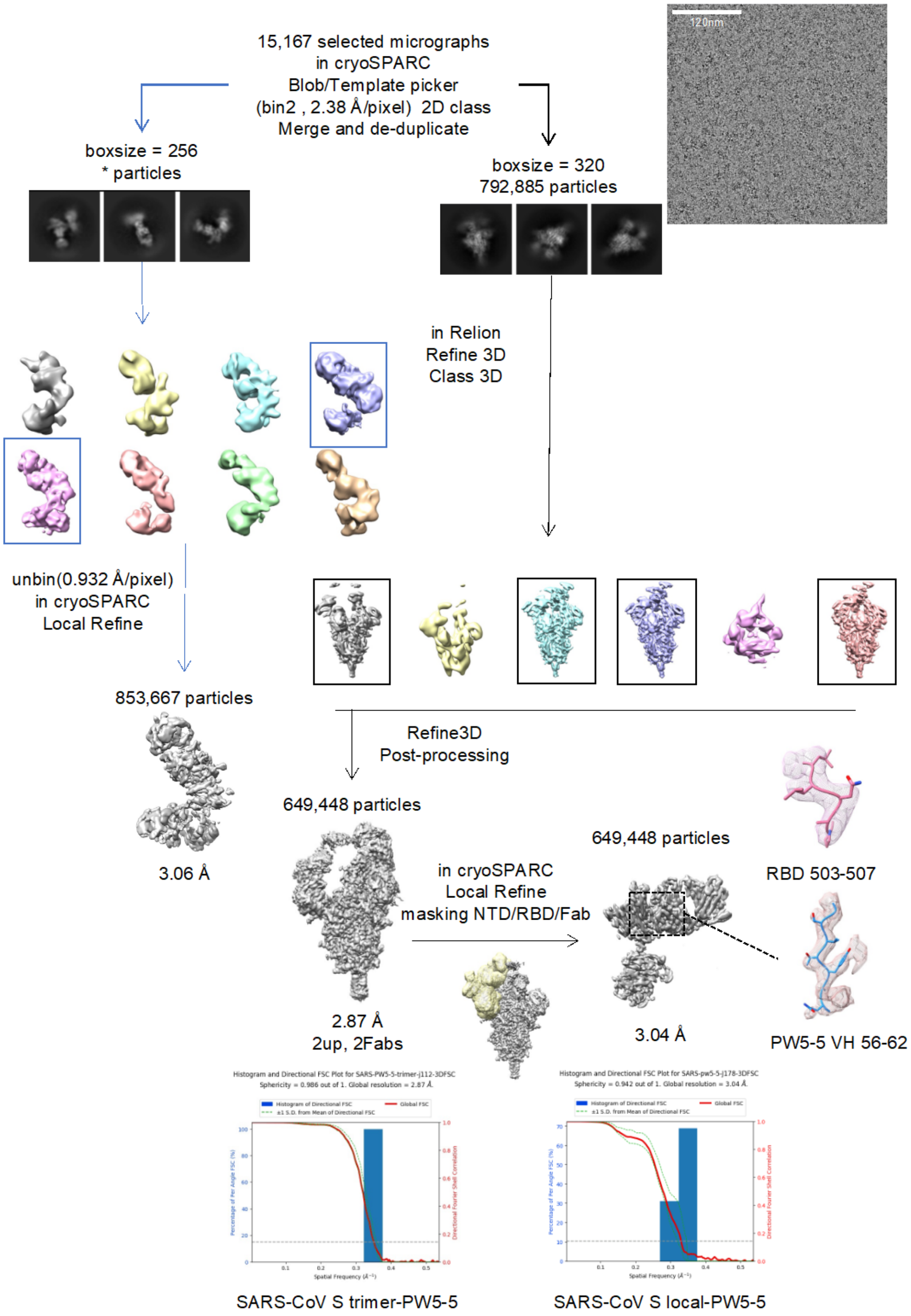

Supplementary Fig. S17 Data processing flowchart of SARS-CoV-S-PW5-5 complex.

Supplementary Fig. S18

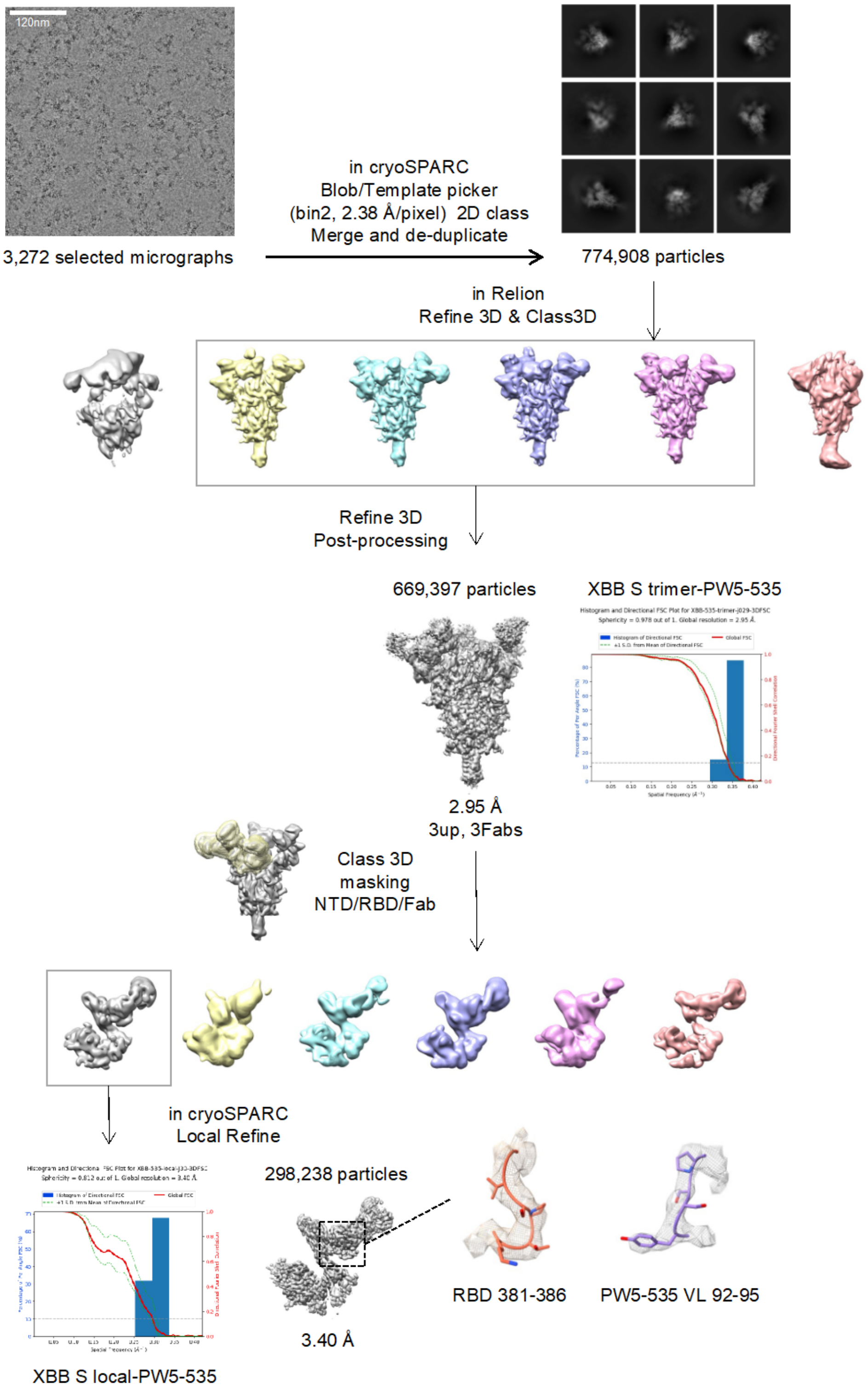

Supplementary Fig. S18 Data processing flowchart of XBB-S-PW5-535 complex.

Supplementary Fig. S19

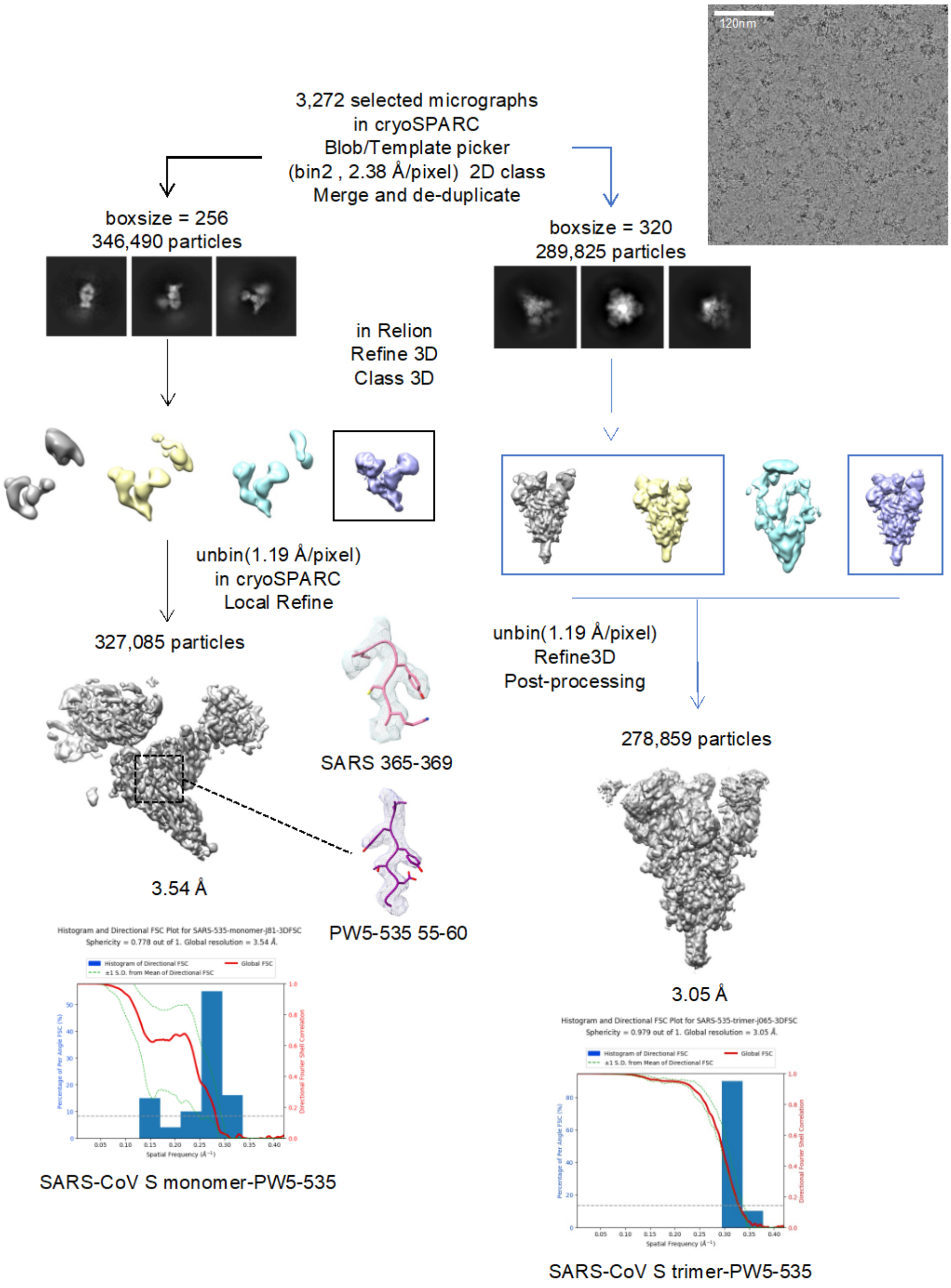

Supplementary Fig. S19 Data processing flowchart of SARS-CoV-S-PW5-535 complex.

Supplementary Table S1 Templates of S trimer used in the modeling.

Trimer type	PDB ID	Resolution	Target
3-RBD-up	7CAK	3.58Å	SARS-CoV-2
2-RBD-up-1-RDB-down	7WOB	3.25 Å	
1-RBD-up-2-RDB-down	7WPA	2.77Å	
3-RBD-down	7OAN	3.0Å	
3-RBD-up	5X59	3.7Å	MERS-CoV
2-RBD-up-1-RDB-down	5X5C	4.10Å	
1-RBD-up-2-RDB-down	5X5F	4.2Å	
3-RBD-down	6Q04	2.8Å	

Supplementary Table S2 Selected antibodies by in-silico screening.

mAb	MERS epitope	Omicron epitope	WT epitope	High-frequency mAb						
	epitope centre	ranking	ranking percentage	epitope centre	ranking	ranking percentage	epitope centre	ranking	ranking percentage	
PW5-6	MERS_3_RBD_down_1821.pdb	4	0.5% ~ 1.0%	omicron_3_RBD_down_1932.pdb	3	0.0% ~ 0.5%	OmiWT_3_RBD_down_1932.pdb	2	0.0% ~ 0.5%	PW5-1
PW5-8	MERS_3_RBD_down_1852.pdb	5	0.5% ~ 1.0%	omicron_3_RBD_down_1963.pdb	7	1.0% ~ 1.5%	OmiWT_3_RBD_down_1963.pdb	2	0.0% ~ 0.5%	PW5-2
PW5-18	MERS_1_RBD_UP_305.pdb	1	0.0% ~ 0.5%	omicron_1_RBD_UP_305.pdb	1	0.0% ~ 0.5%	OmiWT_1_RBD_UP_305.pdb	1	0.0% ~ 0.5%	PW5-4
PW5-19	MERS_3_RBD_down_1315.pdb	8	1.0% ~ 1.5%	omicron_3_RBD_down_1512.pdb	9	1.0% ~ 1.5%	OmiWT_3_RBD_down_1512.pdb	5	0.5% ~ 1.0%	PW5-5
PW5-23	MERS_3_RBD_down_1479.pdb	2	0.0% ~ 0.5%	omicron_3_RBD_down_1691.pdb	2	0.0% ~ 0.5%	OmiWT_3_RBD_down_1691.pdb	2	0.0% ~ 0.5%	PW5-7
PW5-29	MERS_3_RBD_down_1500.pdb	12	1.5% ~ 2.0%	omicron_3_RBD_down_1714.pdb	13	1.5% ~ 2.0%	OmiWT_3_RBD_down_1714.pdb	7	1.0% ~ 1.5%	PW5-10
PW5-207	MERS_3_RBD_down_598.pdb	1	0.0% ~ 0.5%	omicron_3_RBD_down_635.pdb	1	0.0% ~ 0.5%	OmiWT_3_RBD_down_635.pdb	1	0.0% ~ 0.5%	
PW5-212	MERS_3_RBD_UP_2253.pdb	1	0.0% ~ 0.5%	omicron_3_RBD_UP_2524.pdb	1	0.0% ~ 0.5%	OmiWT_3_RBD_UP_2524.pdb	1	0.0% ~ 0.5%	
PW5-233	MERS_3_RBD_down_1315.pdb	1	0.0% ~ 0.5%	omicron_3_RBD_down_1512.pdb	1	0.0% ~ 0.5%	OmiWT_3_RBD_down_1512.pdb	1	0.0% ~ 0.5%	
PW5-238	MERS_3_RBD_down_2293.pdb	5	0.5% ~ 1.0%	omicron_3_RBD_down_2621.pdb	3	0.0% ~ 0.5%	OmiWT_3_RBD_down_2621.pdb	7	1.0% ~ 1.5%	
PW5-240	MERS_3_RBD_down_1815.pdb	10	1.0% ~ 1.5%	omicron_3_RBD_down_1926.pdb	7	1.0% ~ 1.5%	OmiWT_3_RBD_down_1926.pdb	7	1.0% ~ 1.5%	
PW5-241	MERS_2_RBD_UP_887.pdb	5	0.5% ~ 1.0%	omicron_2_RBD_UP_929.pdb	3	0.0% ~ 0.5%	OmiWT_2_RBD_UP_929.pdb	2	0.0% ~ 0.5%	
PW5-253	MERS_3_RBD_down_598.pdb	5	0.5% ~ 1.0%	omicron_3_RBD_down_635.pdb	11	1.5% ~ 2.0%	OmiWT_3_RBD_down_635.pdb	11	1.5% ~ 2.0%	
PW5-259	MERS_3_RBD_down_1745.pdb	12	1.5% ~ 2.0%	omicron_3_RBD_down_1856.pdb	8	1.0% ~ 1.5%	OmiWT_3_RBD_down_1856.pdb	8	1.0% ~ 1.5%	
PW5-265	MERS_3_RBD_down_1884.pdb	9	1.0% ~ 1.5%	omicron_3_RBD_down_1995.pdb	13	1.5% ~ 2.0%	OmiWT_3_RBD_down_1995.pdb	13	1.5% ~ 2.0%	
PW5-270	MERS_3_RBD_UP_2757.pdb	11	1.5% ~ 2.0%	omicron_3_RBD_UP_2858.pdb	4	0.5% ~ 1.0%	OmiWT_3_RBD_UP_2858.pdb	5	0.5% ~ 1.0%	
PW5-274	MERS_3_RBD_down_1791.pdb	12	1.5% ~ 2.0%	omicron_3_RBD_down_1902.pdb	3	0.0% ~ 0.5%	OmiWT_3_RBD_down_1902.pdb	3	0.0% ~ 0.5%	
PW5-283	MERS_3_RBD_down_1852.pdb	6	0.5% ~ 1.0%	omicron_3_RBD_down_1963.pdb	4	0.5% ~ 1.0%	OmiWT_3_RBD_down_1963.pdb	1	0.0% ~ 0.5%	
PW5-288	MERS_3_RBD_down_1479.pdb	10	1.0% ~ 1.5%	omicron_3_RBD_down_1691.pdb	4	0.5% ~ 1.0%	OmiWT_3_RBD_down_1691.pdb	7	1.0% ~ 1.5%	
PW5-298	MERS_3_RBD_down_1458.pdb	9	1.0% ~ 1.5%	omicron_3_RBD_down_1669.pdb	13	1.5% ~ 2.0%	OmiWT_3_RBD_down_1669.pdb	12	1.5% ~ 2.0%	
PW5-313	MERS_3_RBD_down_1479.pdb	11	1.5% ~ 2.0%	omicron_3_RBD_down_1691.pdb	7	1.0% ~ 1.5%	OmiWT_3_RBD_down_1691.pdb	4	0.5% ~ 1.0%	
PW5-331	MERS_3_RBD_UP_2763.pdb	10	1.0% ~ 1.5%	omicron_3_RBD_UP_2864.pdb	11	1.5% ~ 2.0%	OmiWT_3_RBD_UP_2864.pdb	5	0.5% ~ 1.0%	
PW5-335	MERS_3_RBD_down_1489.pdb	3	0.0% ~ 0.5%	omicron_3_RBD_down_1587.pdb	4	0.5% ~ 1.0%	OmiWT_3_RBD_down_1587.pdb	9	1.0% ~ 1.5%	
PW5-363	MERS_3_RBD_UP_2757.pdb	3	0.0% ~ 0.5%	omicron_3_RBD_UP_2858.pdb	5	0.5% ~ 1.0%	OmiWT_3_RBD_UP_2858.pdb	4	0.5% ~ 1.0%	
PW5-380	MERS_3_RBD_UP_2708.pdb	11	1.5% ~ 2.0%	omicron_3_RBD_UP_2809.pdb	7	1.0% ~ 1.5%	OmiWT_3_RBD_UP_2809.pdb	10	1.0% ~ 1.5%	
PW5-394	MERS_3_RBD_down_1881.pdb	4	0.5% ~ 1.0%	omicron_3_RBD_down_1992.pdb	5	0.5% ~ 1.0%	OmiWT_3_RBD_down_1992.pdb	5	0.5% ~ 1.0%	
PW5-399	MERS_2_RBD_UP_744.pdb	5	0.5% ~ 1.0%	omicron_2_RBD_UP_818.pdb	1	0.0% ~ 0.5%	OmiWT_2_RBD_UP_818.pdb	3	0.0% ~ 0.5%	
PW5-412	MERS_3_RBD_down_1851.pdb	1	0.0% ~ 0.5%	omicron_3_RBD_down_1962.pdb	1	0.0% ~ 0.5%	OmiWT_3_RBD_down_1962.pdb	1	0.0% ~ 0.5%	
PW5-419	MERS_3_RBD_down_1812.pdb	6	0.5% ~ 1.0%	omicron_3_RBD_down_1923.pdb	3	0.0% ~ 0.5%	OmiWT_3_RBD_down_1923.pdb	2	0.0% ~ 0.5%	
PW5-420	MERS_3_RBD_down_1812.pdb	12	1.5% ~ 2.0%	omicron_3_RBD_down_1923.pdb	5	0.5% ~ 1.0%	OmiWT_3_RBD_down_1923.pdb	10	1.0% ~ 1.5%	
PW5-421	MERS_3_RBD_down_1500.pdb	8	1.0% ~ 1.5%	omicron_3_RBD_down_1600.pdb	11	1.5% ~ 2.0%	OmiWT_3_RBD_down_1600.pdb	6	0.5% ~ 1.0%	
PW5-428	MERS_3_RBD_down_1817.pdb	6	0.5% ~ 1.0%	omicron_3_RBD_down_1928.pdb	10	1.0% ~ 1.5%	OmiWT_3_RBD_down_1928.pdb	10	1.0% ~ 1.5%	
PW5-432	MERS_3_RBD_UP_1811.pdb	5	0.5% ~ 1.0%	omicron_3_RBD_UP_1888.pdb	13	1.5% ~ 2.0%	OmiWT_3_RBD_UP_1888.pdb	13	1.5% ~ 2.0%	
PW5-433	MERS_3_RBD_down_1506.pdb	12	1.5% ~ 2.0%	omicron_3_RBD_down_1721.pdb	2	0.0% ~ 0.5%	OmiWT_3_RBD_down_1721.pdb	12	1.5% ~ 2.0%	
PW5-436	MERS_3_RBD_down_1823.pdb	9	1.0% ~ 1.5%	omicron_3_RBD_down_1934.pdb	1	0.0% ~ 0.5%	OmiWT_3_RBD_down_1934.pdb	2	0.0% ~ 0.5%	
PW5-459	MERS_3_RBD_UP_619.pdb	5	0.5% ~ 1.0%	omicron_3_RBD_UP_700.pdb	13	1.5% ~ 2.0%	OmiWT_3_RBD_UP_700.pdb	13	1.5% ~ 2.0%	
PW5-460	MERS_3_RBD_down_1872.pdb	13	1.5% ~ 2.0%	omicron_3_RBD_down_1983.pdb	13	1.5% ~ 2.0%	OmiWT_3_RBD_down_1983.pdb	13	1.5% ~ 2.0%	
PW5-468	MERS_3_RBD_down_1827.pdb	2	0.0% ~ 0.5%	omicron_3_RBD_down_1938.pdb	1	0.0% ~ 0.5%	OmiWT_3_RBD_down_1938.pdb	2	0.0% ~ 0.5%	
PW5-477	MERS_1_RBD_UP_60.pdb	10	1.0% ~ 1.5%	omicron_1_RBD_UP_60.pdb	7	1.0% ~ 1.5%	OmiWT_1_RBD_UP_60.pdb	7	1.0% ~ 1.5%	
PW5-479	MERS_1_RBD_UP_60.pdb	5	0.5% ~ 1.0%	omicron_1_RBD_UP_60.pdb	2	0.0% ~ 0.5%	OmiWT_1_RBD_UP_60.pdb	2	0.0% ~ 0.5%	
PW5-492	MERS_3_RBD_UP_1820.pdb	6	0.5% ~ 1.0%	omicron_3_RBD_UP_1897.pdb	7	1.0% ~ 1.5%	OmiWT_3_RBD_UP_1897.pdb	7	1.0% ~ 1.5%	
PW5-494	MERS_3_RBD_down_1851.pdb	2	0.0% ~ 0.5%	omicron_3_RBD_down_1962.pdb	2	0.0% ~ 0.5%	OmiWT_3_RBD_down_1962.pdb	2	0.0% ~ 0.5%	
PW5-508	MERS_3_RBD_down_1670.pdb	11	1.5% ~ 2.0%	omicron_3_RBD_down_1781.pdb	7	1.0% ~ 1.5%	OmiWT_3_RBD_down_1781.pdb	5	0.5% ~ 1.0%	
PW5-510	MERS_1_RBD_UP_1495.pdb	4	0.5% ~ 1.0%	omicron_1_RBD_UP_1739.pdb	4	0.5% ~ 1.0%	OmiWT_1_RBD_UP_1739.pdb	4	0.5% ~ 1.0%	
PW5-522	MERS_3_RBD_UP_2708.pdb	13	1.5% ~ 2.0%	omicron_3_RBD_UP_2809.pdb	3	0.0% ~ 0.5%	OmiWT_3_RBD_UP_2809.pdb	3	0.0% ~ 0.5%	
PW5-524	MERS_3_RBD_UP_2708.pdb	4	0.5% ~ 1.0%	omicron_3_RBD_UP_2809.pdb	8	1.0% ~ 1.5%	OmiWT_3_RBD_UP_2809.pdb	4	0.5% ~ 1.0%	
PW5-530	MERS_3_RBD_down_1376.pdb	5	0.5% ~ 1.0%	omicron_3_RBD_down_1580.pdb	2	0.0% ~ 0.5%	OmiWT_3_RBD_down_1580.pdb	1	0.0% ~ 0.5%	
PW5-534	MERS_3_RBD_down_1799.pdb	13	1.5% ~ 2.0%	omicron_3_RBD_down_1910.pdb	12	1.5% ~ 2.0%	OmiWT_3_RBD_down_1910.pdb	12	1.5% ~ 2.0%	
PW5-535	MERS_1_RBD_UP_847.pdb	3	0.0% ~ 0.5%	omicron_1_RBD_UP_898.pdb	3	0.0% ~ 0.5%	OmiWT_1_RBD_UP_898.pdb	3	0.0% ~ 0.5%	
PW5-540	MERS_3_RBD_down_1568.pdb	3	0.0% ~ 0.5%	omicron_3_RBD_down_1673.pdb	6	0.5% ~ 1.0%	OmiWT_3_RBD_down_1673.pdb	6	0.5% ~ 1.0%	
PW5-544	MERS_1_RBD_UP_1640.pdb	1	0.0% ~ 0.5%	omicron_1_RBD_UP_1775.pdb	1	0.0% ~ 0.5%	OmiWT_1_RBD_UP_1775.pdb	2	0.0% ~ 0.5%	
PW5-546	MERS_3_RBD_down_1479.pdb	1	0.0% ~ 0.5%	omicron_3_RBD_down_1691.pdb	1	0.0% ~ 0.5%	OmiWT_3_RBD_down_1691.pdb	1	0.0% ~ 0.5%	
PW5-547	MERS_3_RBD_down_1315.pdb	6	0.5% ~ 1.0%	omicron_3_RBD_down_1512.pdb	6	0.5% ~ 1.0%	OmiWT_3_RBD_down_1512.pdb	10	1.0% ~ 1.5%	
PW5-558	MERS_3_RBD_down_1535.pdb	8	1.0% ~ 1.5%	omicron_3_RBD_down_1751.pdb	4	0.5% ~ 1.0%	OmiWT_3_RBD_down_1751.pdb	4	0.5% ~ 1.0%	
PW5-570	MERS_3_RBD_down_1284.pdb	3	0.0% ~ 0.5%	omicron_3_RBD_down_1479.pdb	6	0.5% ~ 1.0%	OmiWT_3_RBD_down_1479.pdb	6	0.5% ~ 1.0%	
PW5-576	MERS_1_RBD_UP_1574.pdb	3	0.0% ~ 0.5%	omicron_1_RBD_UP_1822.pdb	1	0.0% ~ 0.5%	OmiWT_1_RBD_UP_1822.pdb	1	0.0% ~ 0.5%	
PW5-609	MERS_1_RBD_UP_60.pdb	7	1.0% ~ 1.5%	omicron_1_RBD_UP_60.pdb	3	0.0% ~ 0.5%	OmiWT_1_RBD_UP_60.pdb	3	0.0% ~ 0.5%	
PW5-633	MERS_3_RBD_UP_1820.pdb	1	0.0% ~ 0.5%	omicron_3_RBD_UP_1897.pdb	1	0.0% ~ 0.5%	OmiWT_3_RBD_UP_1897.pdb	1	0.0% ~ 0.5%	
PW5-646	MERS_3_RBD_UP_1820.pdb	8	1.0% ~ 1.5%	omicron_3_RBD_UP_1897.pdb	9	1.0% ~ 1.5%	OmiWT_3_RBD_UP_1897.pdb	9	1.0% ~ 1.5%	
PW5-656	MERS_3_RBD_down_1794.pdb	7	1.0% ~ 1.5%	omicron_3_RBD_down_1905.pdb	8	1.0% ~ 1.5%	OmiWT_3_RBD_down_1905.pdb	6	0.5% ~ 1.0%	
PW5-676	MERS_3_RBD_down_1851.pdb	5	0.5% ~ 1.0%	omicron_3_RBD_down_1962.pdb	8	1.0% ~ 1.5%	OmiWT_3_RBD_down_1962.pdb	7	1.0% ~ 1.5%	
PW5-683	MERS_3_RBD_down_1793.pdb	4	0.5% ~ 1.0%	omicron_3_RBD_down_1904.pdb	1	0.0% ~ 0.5%	OmiWT_3_RBD_down_1904.pdb	7	1.0% ~ 1.5%	
PW5-693	MERS_1_RBD_UP_1495.pdb	9	1.0% ~ 1.5%	omicron_1_RBD_UP_1739.pdb	3	0.0% ~ 0.5%	OmiWT_1_RBD_UP_1739.pdb	3	0.0% ~ 0.5%	
PW5-694	MERS_3_RBD_down_1284.pdb	1	0.0% ~ 0.5%	omicron_3_RBD_down_1479.pdb	4	0.5% ~ 1.0%	OmiWT_3_RBD_down_1479.pdb	4	0.5% ~ 1.0%	
PW5-699	MERS_3_RBD_down_1884.pdb	8	1.0% ~ 1.5%	omicron_3_RBD_down_1995.pdb	12	1.5% ~ 2.0%	OmiWT_3_RBD_down_1995.pdb	12	1.5% ~ 2.0%	
PW5-710	MERS_3_RBD_UP_2253.pdb	8	1.0% ~ 1.5%	omicron_3_RBD_UP_2524.pdb	2	0.0% ~ 0.5%	OmiWT_3_RBD_UP_2524.pdb	2	0.0% ~ 0.5%	
PW5-720	MERS_3_RBD_down_1500.pdb	2	0.0% ~ 0.5%	omicron_3_RBD_down_1600.pdb	4	0.5% ~ 1.0%	OmiWT_3_RBD_down_1600.pdb	9	1.0% ~ 1.5%	
PW5-722	MERS_3_RBD_UP_619.pdb	10	1.0% ~ 1.5%	omicron_3_RBD_UP_700.pdb	13	1.5% ~ 2.0%	OmiWT_3_RBD_UP_700.pdb	13	1.5% ~ 2.0%	
PW5-786	MERS_1_RBD_UP_1640.pdb	13	1.5% ~ 2.0%	omicron_1_RBD_UP_1775.pdb	5	0.5% ~ 1.0%	OmiWT_1_RBD_UP_1775.pdb	10	1.0% ~ 1.5%	
PW5-803	MERS_1_RBD_UP_60.pdb	4	0.5% ~ 1.0%	omicron_1_RBD_UP_60.pdb	4	0.5% ~ 1.0%	OmiWT_1_RBD_UP_60.pdb	4	0.5% ~ 1.0%	
PW5-807	MERS_1_RBD_UP_1640.pdb	10	1.0% ~ 1.5%	omicron_1_RBD_UP_1775.pdb	10	1.0% ~ 1.5%	OmiWT_1_RBD_UP_1775.pdb	9	1.0% ~ 1.5%	
PW5-808	MERS_3_RBD_down_1881.pdb	8	1.0% ~ 1.5%	omicron_3_RBD_down_1992.pdb	7	1.0% ~ 1.5%	OmiWT_3_RBD_down_1992.pdb	7	1.0% ~ 1.5%	
PW5-819	MERS_3_RBD_down_1740.pdb	1	0.0% ~ 0.5%	omicron_3_RBD_down_1851.pdb	3	0.0% ~ 0.5%	OmiWT_3_RBD_down_1851.pdb	3	0.0% ~ 0.5%	
PW5-850	MERS_1_RBD_UP_60.pdb	1	0.0% ~ 0.5%	omicron_1_RBD_UP_60.pdb	1	0.0% ~ 0.5%	OmiWT_1_RBD_UP_60.pdb	1	0.0% ~ 0.5%	
PW5-853	MERS_3_RBD_UP_1811.pdb	6	0.5% ~ 1.0%	omicron_3_RBD_UP_1888.pdb	6	0.5% ~ 1.0%	OmiWT_3_RBD_UP_1888.pdb	6	0.5% ~ 1.0%	
PW5-862	MERS_1_RBD_UP_305.pdb	4	0.5% ~ 1.0%	omicron_1_RBD_UP_305.pdb	9	1.0% ~ 1.5%	OmiWT_1_RBD_UP_305.pdb	9	1.0% ~ 1.5%	
PW5-879	MERS_1_RBD_UP_60.pdb	8	1.0% ~ 1.5%	omicron_1_RBD_UP_60.pdb	11	1.5% ~ 2.0%	OmiWT_1_RBD_UP_60.pdb	11	1.5% ~ 2.0%	
PW5-884	MERS_1_RBD_UP_60.pdb	6	0.5% ~ 1.0%	omicron_1_RBD_UP_60.pdb	8	1.0% ~ 1.5%	OmiWT_1_RBD_UP_60.pdb	8	1.0% ~ 1.5%	
PW5-885	MERS_1_RBD_UP_60.pdb	3	0.0% ~ 0.5%	omicron_1_RBD_UP_60.pdb	13	1.5% ~ 2.0%	OmiWT_1_RBD_UP_60.pdb	13	1.5% ~ 2.0%	
PW5-897	MERS_1_RBD_UP_305.pdb	11	1.5% ~ 2.0%	omicron_1_RBD_UP_305.pdb	4	0.5% ~ 1.0%	OmiWT_1_RBD_UP_305.pdb	4	0.5% ~ 1.0%	

Supplementary Table S3 Cryo-EM data collection and refinement statistics.

	PW5-5					PW5-535				PW5-570	
	XBB			SARS-CoV		XBB		SARS-CoV		BA.1	
	timer-1 3up,3Fabs	trimer-2, 2up,2Fabs	monomer	trimer	local	trimer	local	trimer	monomer	trimer	local
**Data collection and processing**											
Magnification			130,000					105,000			
Voltage (kV)			300					300			
Total dose (e–/Å^2)			50					50			
Defocus range (μm)			-1.0 to -3.0					-1.0 to -3.0			
Pixel size (Å)			0.932					1.19			
Symmetry imposed			C1					C1			
Final particles (no.)	208,476	130,701	166,548	649,448	649,448	669,397	298,238	278,859	327,085	439,708	439,708
Map resolution (Å)	3.14	3.21	3.73	2.87	3.04	2.95	3.40	3.05	3.54	2.78	2.93
**Refinement**											
**R.m.s. deviations**											
Bond lengths (Å)	0.002	0.002	0.003	0.002	0.002	0.002	0.002	0.002	0.002	0.002	0.002
Bond angles (°)	0.516	0.484	0.653	0.448	0.515	0.469	0.619	0.470	0.478	0.437	0.638
**Validation**											
MolProbity score	2.22	2.17	2.46	2.33	2.44	2.24	2.57	2.36	2.65	1.97	2.09
Clashscore	6.38	6.27	7.21	7.95	7.77	6.77	8.73	6.91	9.40	5.38	4.63
Rotamer outlier (%)	4.46	4.27	5.75	4.45	5.91	4.76	6.20	5.05	5.79	3.03	2.78
**Ramachandran**											
**plot**	94.48	94.93	92.11	94.01	93.38	94.90	91.57	92.97	89.18	95.43	91.07
Favored (%)	5.52	5.04	7.70	5.99	6.62	5.08	8.43	6.96	10.71	4.57	8.93
Allowed (%)	0.00	0.02	0.19	0.00	0.00	0.02	0.00	0.07	0.10	0.00	0.00
Disallowed (%)											
**EMDB**	EMD-37164	EMD-37157	EMD-37160	EMD-37139	EMD-37145	EMD-37165	EMD-37143	EMD-37144	EMD-37161	EMD-37162	EMD-37163
**PDB**	8KEQ	8KEH	8KEJ	8KDM	8KDT	8KER	8KDR	8KDS	8KEK	8KEO	8KEP
